# ^13^C- and ^15^N-Labeling Strategies Combined with Mass Spectrometry Comprehensively Quantify Phospholipid Dynamics in *C*. *elegans*

**DOI:** 10.1371/journal.pone.0141850

**Published:** 2015-11-03

**Authors:** Blair C. R. Dancy, Shaw-Wen Chen, Robin Drechsler, Philip R. Gafken, Carissa Perez Olsen

**Affiliations:** 1 Division of Basic Sciences, Fred Hutchinson Cancer Research Center, Seattle, Washington, United States of America; 2 Proteomics Facility, Fred Hutchinson Cancer Research Center, Seattle, Washington, United States of America; University of Geneva, SWITZERLAND

## Abstract

Membranes define cellular and organelle boundaries, a function that is critical to all living systems. Like other biomolecules, membrane lipids are dynamically maintained, but current methods are extremely limited for monitoring lipid dynamics in living animals. We developed novel strategies in *C*. *elegans* combining ^13^C and ^15^N stable isotopes with mass spectrometry to directly quantify the replenishment rates of the individual fatty acids and intact phospholipids of the membrane. Using multiple measurements of phospholipid dynamics, we found that the phospholipid pools are replaced rapidly and at rates nearly double the turnover measured for neutral lipid populations. In fact, our analysis shows that the majority of membrane lipids are replaced each day. Furthermore, we found that stearoyl-CoA desaturases (SCDs), critical enzymes in polyunsaturated fatty acid production, play an unexpected role in influencing the overall rates of membrane maintenance as SCD depletion affected the turnover of nearly all membrane lipids. Additionally, the compromised membrane maintenance as defined by LC-MS/MS with *SCD* RNAi resulted in active phospholipid remodeling that we predict is critical to alleviate the impact of reduced membrane maintenance in these animals. Not only have these combined methodologies identified new facets of the impact of SCDs on the membrane, but they also have great potential to reveal many undiscovered regulators of phospholipid metabolism.

## Introduction

Despite constant movement of membrane components, the appropriate lipid compositions must be maintained as membranes are not static barriers that simply encapsulate cells and their organelles. In fact, each membrane within a cell maintains a unique lipid composition that is optimized for membrane function since the makeup of the membrane influences its permeability, fluidity, and curvature [1, 2]. In turn, the biophysical properties of the membrane impact basic cellular processes including the function of membrane proteins, efficient vesicle formation and even which molecules enter and exit the cell [3, 4]. In addition to influencing normal cellular function, aberrant membrane structure has been observed in numerous diseases including cancers and neurodegenerative diseases [5, 6]. Moreover, altered membrane composition itself can explain ineffective drug delivery in cancer cells, highlighting the importance of understanding how the membrane is defined [7].

The lipids provided to the membrane must be carefully regulated as any given membrane contains more than 600 distinct phospholipid (PL) species [1, 8]. Generally, these PLs contain a glycerol molecule with a polar headgroup at the *sn-3* position and two acyl chains at the *sn-1* and *sn-2* positions. Much of the diversity in PLs is generated through variance in either the headgroups, most commonly choline and ethanolamine, or in the incorporation of different fatty acids (FAs), from saturated to highly polyunsaturated chains [8]. Although the regulatory mechanisms have not been established, many of the enzymatic pathways that generate the lipids for the membrane have been defined. The new FA moieties provided to the membrane can be directly derived from the diet or generated through *de novo* FA synthesis [9]. Regardless of the origin of the new fatty acids, FA desaturases and elongases participate in manufacturing the diverse FA species provided to phospholipids [10]. The FAs produced through the elongation and desaturation pathway can be directly incorporated into the bilayer via acyltransferase activity or funneled into PL synthesis pathways.

There is very little known about the mechanisms that sense the types of lipids needed for membrane maintenance and orchestrate their provision; however, the FA synthesis pathway has emerged as a convergence point for multiple events that may modulate membrane homeostasis and adaptation. In particular, stearoyl-CoA desaturases (SCDs) introduce the first degree of unsaturation into a stearoyl-CoA molecule (C18:0), and SCDs have a clear role in regulating lipid composition, as their knockdown results in an increase in saturated fats in species ranging from *C*. *elegans* to mice [11, 12]. The dysregulation of SCDs in humans has been directly implicated in certain cancers and obesity [13, 14]. Furthermore, the *SCD* genes are tightly regulated and respond to changes in diet, hormonal signals and environmental cues, illustrating their impact on membrane composition and adaptation [15–17]. Although the role of these genes in day-to-day membrane turnover has not been explored, there are many indications that *SCD* genes may coordinate membrane dynamics. In *C*. *elegans*, there are two *SCD* genes, *fat-6* and *fat-*7, and, through the use of double mutant strains, these genes have been shown to influence PL headgroup distribution, lipid droplet formation, and FA composition [18, 19]. Additionally, there is an accumulation of atypical FA species in the membrane in these *fat-6;fat-7* animals, further supporting a role for the SCD genes in other aspects of membrane preservation [11].

Membrane lipids are constantly consumed by normal cellular processes including intracellular trafficking, β-oxidation, exocytosis and even damage; in order to preserve cellular compartmentalization, there must be a continual replacement of membrane lipids, a process that we refer to as “membrane maintenance”. The abundant interactions of intracellular membranes via direct contacts or through vesicle pathways is evident; however, the biochemical characterization of membrane flux has been more limited [20]. Previous quantification of membrane dynamics has been restricted by the need to incorporate exogenously provided radiolabeled or stable isotope-labeled tracer lipids, limiting the measurements to select molecules [21, 22]. Because the membrane is built of hundreds of different lipid species, monitoring the turnover of a few lipids provides an incomplete picture of global membrane dynamics. Past studies have also been hindered by insufficient enrichment of isotopes, resulting in the absence of a viable animal model for high-throughput interrogation of the genes and pathways that regulate membrane composition and maintenance.

The nematode has the advantage of being amenable to stable isotope labeling of the entire diet, allowing for the incorporation of a general tracer to map lipid metabolism pathways without introducing major changes to the standard laboratory diet [9, 23, 24]. Furthermore, advances in mass spectrometry, particularly electrospray ionization coupled with liquid chromatography, have increased the capacity to measure the vast array of lipids within the membrane [25, 26]. Here, we introduce stable isotope tracers to monitor membrane dynamics at the levels of the individual FA constituents and the intact PLs. We demonstrate how these tools can thoroughly quantify overall membrane replacement and define the unique turnover of individual membrane lipids. The mapping of basal membrane maintenance can then be applied to understand how the membrane responds to major perturbations, such as those seen in human diseases where FA production is compromised through SCD inhibition. We reveal a new role for SCDs in impacting the rates of phospholipid replacement and, in doing so, outline the tools needed to understand the regulation of membrane dynamics.

## Results

### Quantification of the Dynamics of Individual Membrane Fatty Acids with ^13^C-Labeling

Membranes are highly dynamic structures; however, there is no current methodology to fully map the movements of the phospholipid population. The challenge to probing membrane dynamics is that there are many distinct FA species that are found in different quantities. Therefore, a method is needed that can simultaneously track the movement of all the individual FA tails in and out of the membrane. Thus, we fed animals a ^13^C-enriched diet, as our previous work demonstrated that the ^13^C label can be detected in the major FA species [9, 24]. Additionally, the use of dietary ^13^C has the advantages of allowing the animals to eat their standard *E*. *coli* (OP50) laboratory diet and ensuring the incorporation of isotopes in any molecules containing carbon including other lipid populations such as neutral lipids (NLs).

In order to exclusively measure the day-to-day maintenance of the membrane, post-reproductive day 3 sterile adults, *fer-15(b26);fem-1(hc17)*, were selected as our control population. These animals have an intact reproductive tract but lay unfertilized eggs, a feature that allows us to isolate adult populations without impacting the physiological demands of reproduction. Due to their rapid growth, larval animals have different metabolic requirements that would ultimately confound the measurements of adult membrane dynamics. To limit the impact of reproduction on metabolism, we grew nematodes to adulthood on unlabeled *E*.*coli* and then transferred the animals to prepared stable isotope feeding plates at day 3 of adulthood after the majority of eggs have been laid. Taken together, the *fer-15;fem-1* animals allow us to specifically measure membrane maintenance and to limit the impact of life stage, reproduction, and growth on membrane metabolism.

**Fig 1:**
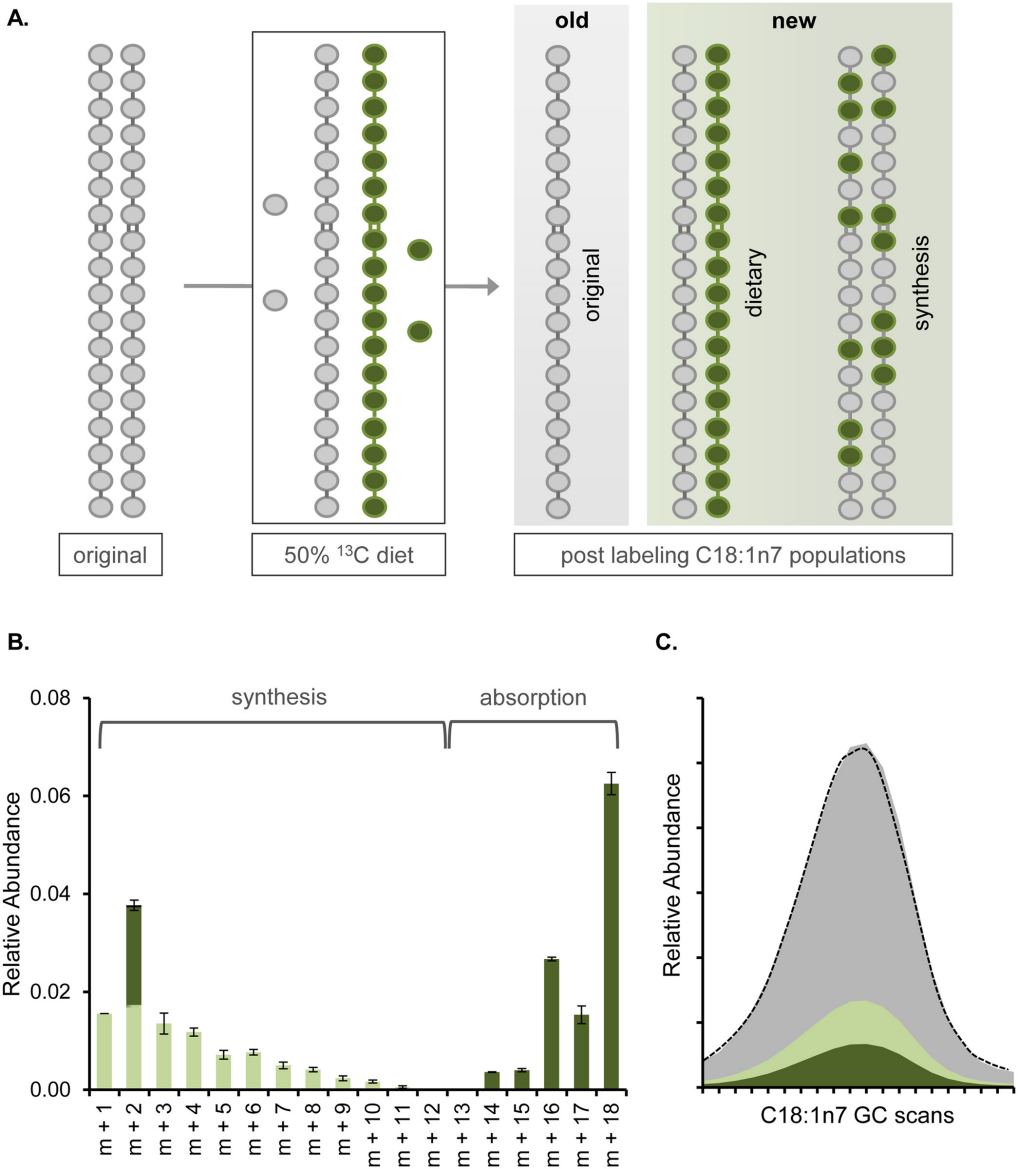
Dietary ^13^C Allows for Modeling of Individual FA Dynamics. (A) Day 3 adult nematodes are grown on ^12^C (gray)-*E*.*coli* (OP50) before the diet is switched to 50% ^13^C (green)-*E*. *coli*. After 6 hours, the C18:1n7 in the nematode was a combination of old fat from the original (pre-^13^C) diet, FA absorbed directly from the diet, and FA derived from lipogenesis with a random but statistically definable incorporation of single carbon molecules. (B) The C18:1n7 isotopomers were assessed by GC-MS. The natural abundance of ^13^C in the environment as well as background were subtracted from the C18:1n7 isotopomers derived from ^13^C-fed animals. The contributions of *de novo* FA synthesis (light green) and dietary absorption (dark green) can be mathematically determined (see Material and Methods, [9]). (C) For any given fatty acid species including the C18:1n7 shown here, the total abundance of the fatty acid is determined by integrating the area under its peak in the gas chromatograph (total abundance outlined in dashed line). Further, we can divide the area as follows with gray representing ^12^C species and green indicating the presence of at least one ^13^C molecule. The unlabeled fatty acids absorbed during the labeling period were accounted for as new. The percentage of the population from absorption (dark green) and synthesis (light green) can be quantified to ultimately define that 29.3 ± 1.5% of the C18:1n7 peak is generated from new fatty acid.

We first considered the incorporation of dietary carbon into the single largest contributor of the young adult membrane, vaccenic acid (C18:1n7 using CX:YnZ nomenclature where X indicates the number of carbons, Y represents the number of double bonds and Z denotes the position of the double bonds) and used gas chromatography and mass spectrometry (GC-MS) to probe the dynamics of this population. Synchronized larval animals were grown until day 3 of adulthood when they were transferred to a 1:1 mixture of ^12^C and ^13^C-*E*. *coli* to introduce the ^13^C-label to quantify the accumulation of new carbon in the fatty acids tails. By feeding adults the ^13^C-labeled diet for 6 hours, the population of dietary fatty acids were either fully ^12^C or fully ^13^C after accounting for the natural abundance of ^13^C in the environment and impurities in the ^13^C media. In *E*. *coli* grown in the absence of nematodes, there are only fully ^12^C- or ^13^C-fatty acids, indicating that no mixing of carbons occurs between the bacterial populations (see S1 Fig). Likewise, the acetyl-CoA units used in *de novo* fatty acid synthesis contained both unlabeled (^12^C-^12^C) and labeled (^13^C-^13^C) populations. There are also small amounts of mixed acetyl-CoA molecules (^12^C-^13^C) that originate from the reassembly of acetyl-CoA after digestion of both bacterial populations [9]. We saw the accumulation of intact dietary FA as well as the incorporation of individual ^13^C-tracers into FAs via lipogenesis, both of which were considered “new” to the membrane (Fig 1A).

By examining the mass spectra for the C18:1n7 population for purified nematode PLs, we detected all potential molecular weights or isotopomers, ranging from 1 isotope incorporation event (m+1) to fully isotopically labeled FAs (m+18) (Fig 1B). After subtraction of the natural abundance of ^13^C isotopes in the environment, the pattern of isotopomers can be explained by considering the following sources: (1) original FA (m+0), (2) *de novo* FA synthesis and other single ^13^C-incorporation events (m+1, m+2…m+13), and (3) incorporation of dietary C18:0 (m+0:m+2 and m+14:m+18) (Fig 1B) [9]. Because the diet is a 1:1 ratio of ^12^C-*E*. *coli* and ^13^C-*E*. *coli*, there is new dietary fat that does not contain any stable isotope and is therefore indistinguishable from the original FAs in the membrane; however, this population can be inferred from the absorption of fully ^13^C-FA as we can determine the exact composition of the *E*. *coli* diet (see S1 Fig). The total absorbed FA population and the ^13^C-FAs derived from *de novo* lipogenesis reveal that 29.3 ± 2.5% of the C18:1n7 population in the membrane is newly incorporated in the 6 hour labeling period (Fig 1C). In summary, dietary stable isotopes can be effectively applied to quantify the dynamics of a single FA population without disruption of the natural diet through exogenously provided lipids.

### Distinct Dynamics of Individual Membrane Fatty Acids

**Fig 2:**
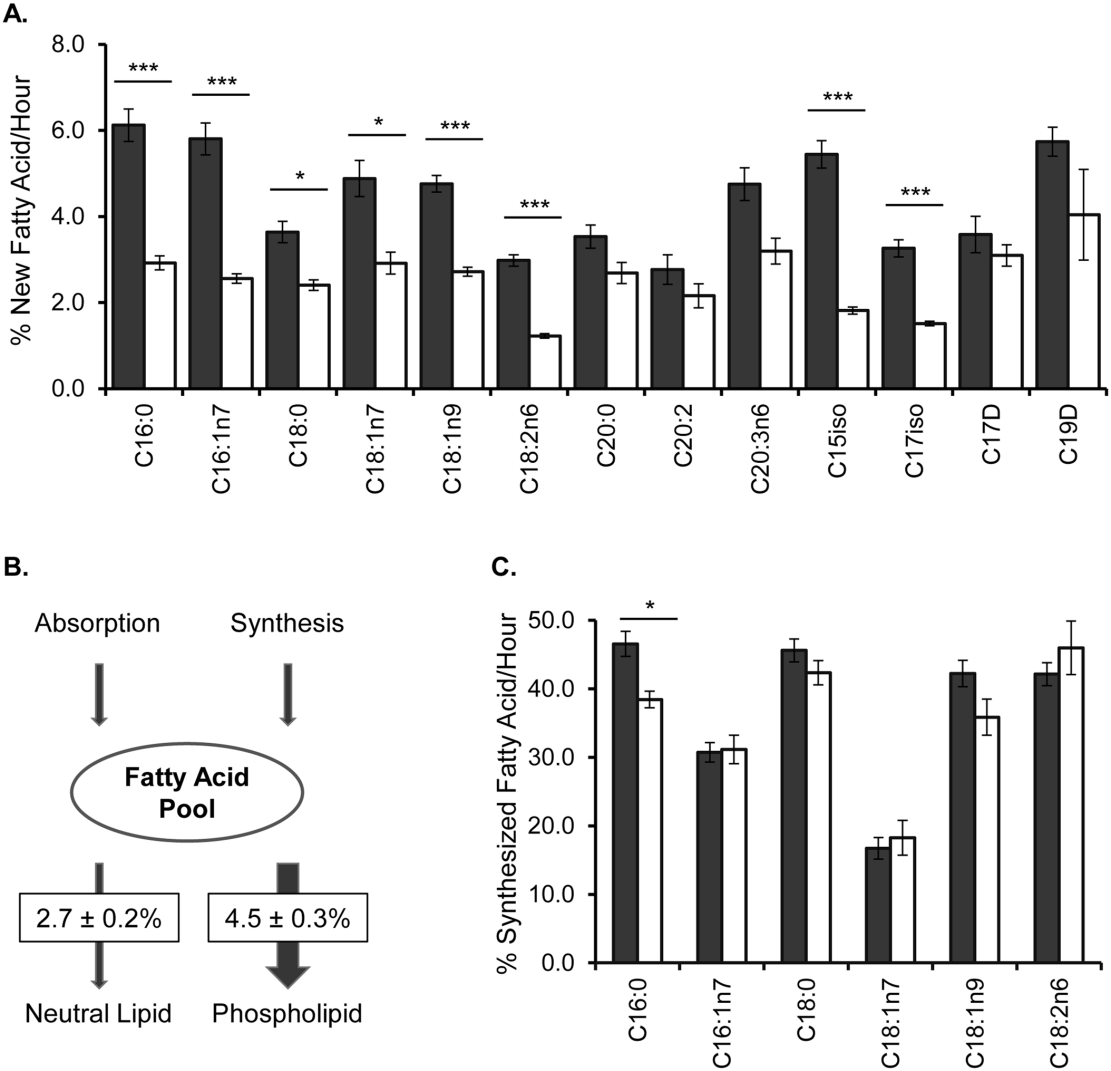
Quantification of Membrane Dynamics With ^13^C-Labeling and GC-MS in Young Adults. (A) Day 3 adult nematodes were fed a diet of 50% ^13^C-labeled *E*. *coli* for 6 hours to introduce ^13^C into their lipids and mark them as newly added or modified. The ^13^C can be traced into the major membrane FAs (>2% abundance) by GC-MS (see S1 Table). The amount of new FA measured by ^13^C-incorporation, was determined for purified PLs (black) and NLs (white). C20s with 4 or more double bonds were excluded in the analysis due to insufficient isotopomers. C20:0 and C20:2 FAs were profiled despite low abundance in the membrane to increase the representation of long-chain FAs. (B) After combining the amount of new FA found in all major C15 to C19 FAs, the overall contribution of new FA/hour was significantly greater in the PLs versus the NLs. (C) The relative amount of new FA derived from *de novo* FA synthesis was not significantly different for PLs (black) and NLs (white) except for C16:0. Numbers shown represent the mean of at least four experiments ± SEM. Statistical significance was calculated by two-tailed unpaired t-tests where significance (*p<0.05, **p<0.01, ***p<0.001) is noted by the asterisks.

As our goal is to monitor multiple species in parallel, we took advantage of the enrichment of the ^13^C label and extended our analysis to the other FAs of the membrane. We could successfully and simultaneously monitor more than 75% of membrane FAs. The only notable exceptions were the C20:4 and C20:5 polyunsaturated fatty acids (PUFAs) that extensively fragment in the mass spectrometer. The rates of membrane FA replacement ranged from 2.8 ± 0.3% new C18:2n6 per hour to 6.1 ± 0.4% new C16:0 per hour (Fig 2A). To assess the validity of using *fer-15;fem-1* animals to model fatty acid dynamics, we compared the rates of ^13^C-incorporation into the phospholipids in wild-type N2 worms, and, although an accurate comparison cannot be done at day 3, we did not see significant differences at day 1 of adulthood, suggesting that *fer-15;fem-1* animals can be used to measure wild-type membrane metabolism (S2 Fig). The differences in the new FA incorporation from *fer-15;fem-1* nematodes emphasized the importance of multiple measurements of membrane flux as even highly related species have distinct replacement rates. These findings suggest that modeling membrane dynamics with a single tracer lipid is not necessarily indicative of overall membrane maintenance. We show that using a ^13^C-enriched diet allows for the monitoring of the majority of the membrane FAs in the same animals, laying the foundation for a more comprehensive understanding of membrane biology.

The replacement rates of the fatty acid populations of the membrane predicted a total rejuvenation of the membrane after 24 hours. In order to test this prediction, we labeled nematodes with stable isotopes for a full 24-hour period and determined the amount of new fatty acid present (see S3 Fig). Indeed, we quantify abundant turnover in the longer feeding period, supporting the fast dynamics defined by the 6-hour labeling period. In some species like C18:0 and C18:2n6, there is a good alignment between the predictions and the measured data. In other species like C16:0, the hourly replacement rate suggested complete rejuvenation of the fatty acid pool within a day; however, the 24-hour labeling found only 66.5 ± 2.5% replacement (S3 Fig). The longer labeling period demonstrates that, even though the majority of the membrane is new within 24 hours, there is a stable population of fatty acids that is protected, perhaps by their location in specific membrane domains.

### Fatty Acids are Preferentially Allocated to Phospholipid Maintenance

In addition to the polar PLs, FAs are critical components of neutral fat storage lipids mainly in the form of triacylglycerols. Although the distributions of the FAs are different, the same FA species are found in PLs and NLs (see S1 Table), allowing us to directly compare the dynamics of these populations after separating the classes by solid phase extraction. In all FA species, there is significantly less new fat associated with NL (Fig 2A). We examined the total impact that FA replenishment has on the overall PL and NL populations by considering the abundance of each FA species in each lipid class. Specifically, we used ^13^C labeling to define how much of an individual FA peak was generated by new FA and found that 4.5 ± 0.3% of the total membrane was replaced with new fat each hour while only 2.7 ± 0.2% of NLs were replaced with new FA each hour (Fig 2B). The stark asymmetry in the FA replacement between these two populations demonstrates that, in young adults, maintenance of the membrane is paramount to the building of fat stores.

### Dietary and Synthesized Fatty Acids are Both Major Contributors to Membrane Maintenance

The stable isotope feeding approach implemented here traces new carbon within the PLs; moreover, the patterns of isotope incorporation are unique depending on the origin of the new FAs (see Material and Methods and Fig 1A). Dietary fat absorption and *de novo* FA synthesis both contribute to the FA pools in animals, and a mixture of ^12^C- and ^13^C-*E*. *coli* has previously determined that relative FA synthesis is responsible for approximately 5% of the total C18:1n7 population in larval animals [9]. In day 3 adults, the proportion of synthesized fat was considerably higher, at 16.7 ± 1.6%, for C18:1n7 than seen in larval (L4) stage animals, indicating an increased reliance on FA synthesis in adult animals (Fig 2C). Here, we looked for the source of the major FAs (>2% of the membrane) that were provided for membrane maintenance and fat stores. In doing so, we found significant contributions from both fat absorption and synthesis. There was no statistically significant difference between PLs and NLs, indicating that FAs pool are not selectively allocated to either PLs or NLs based on whether they were generated from lipogenesis or absorption (Fig 2C). The reduced relative contribution of synthesized fat to C18:1n7 is likely due to the significant presence of that FA in the diet where it comprises ~16% of the total dietary FAs. Previous studies have indicated that the monomethyl-branched chain fatty acids (mmBCFAs), C15iso and C17iso, are entirely synthesized, while the cyclopropyl fats, C17D and C19D are exclusively provided by the diet, thus these FAs were not included here [9, 27]. Taken together, the stable isotope feeding approach has revealed significant turnover of PL populations, with FAs principally obtained directly from the diet and, to a slightly lesser extent, *de novo* FA synthesis.

### Adult-Only RNAi Identifies Genes That Impact the Rates of Fatty Acid Replacement in the Membrane

**Fig 3:**
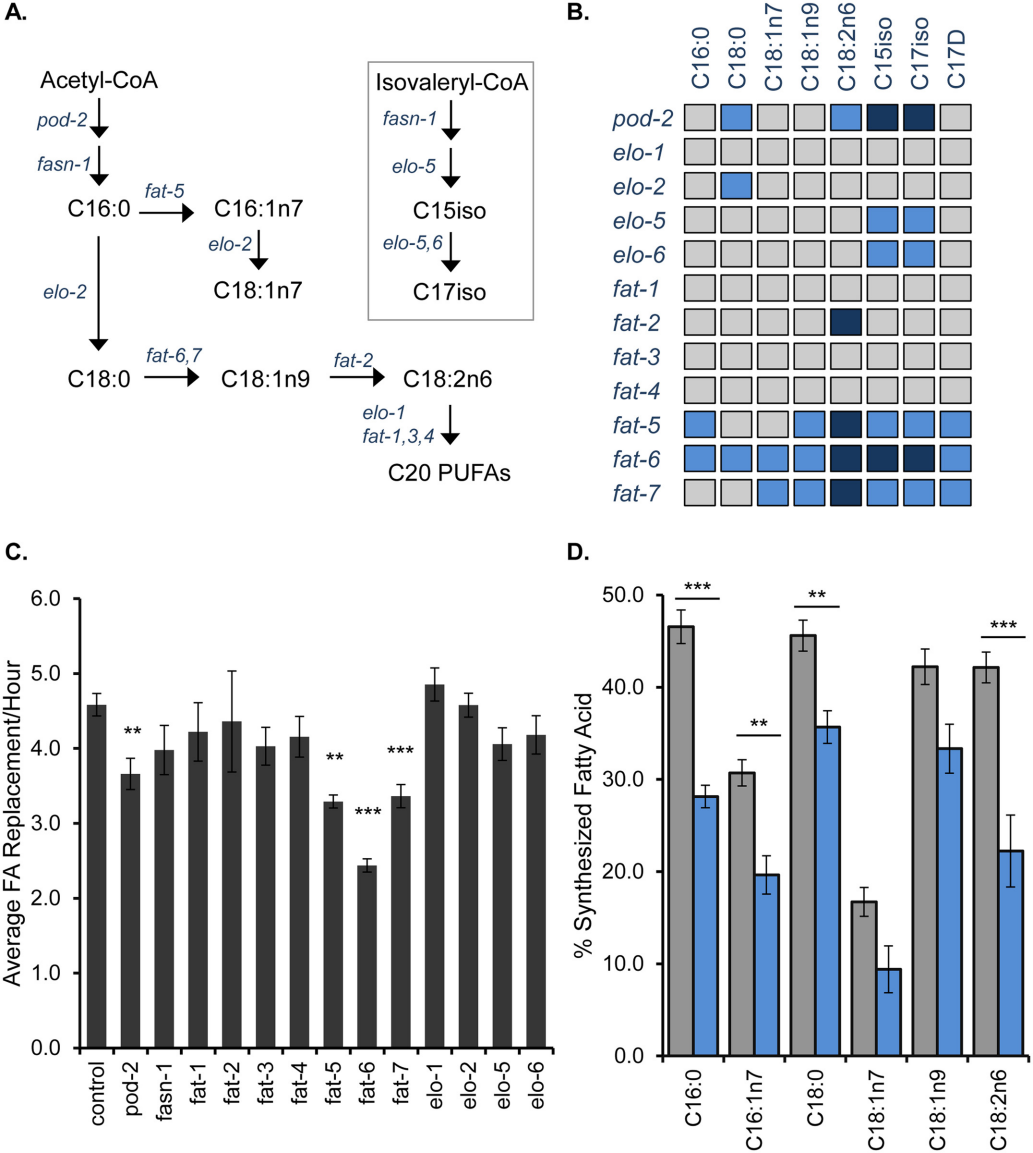
Characterization of SCDs as Membrane Maintenance Regulators. (A) The major FAs in *C*. *elegans* are produced by the elongation and desaturation pathway shown here. An alternate pathway downstream of FA synthase *(fasn-1)* is used for the production of monomethyl-branched chain FAs, C15iso and C17iso. (B) The amount of new FA incorporated each hour was quantified in animals treated with adult-only RNAi against the FA synthesis, elongation and desaturation genes for 48 hours. The resulting data are summarized here with species with less replenishment than control RNAi colored light blue (>25% decrease) and dark blue (>50% decrease). See S1 Dataset for complete source data. (C) To determine the overall impact on membrane replenishment, we averaged the replacement rates for each FA species and normalized by the abundance of each species in the PL population. RNAi of *pod-2*, *fat-5*, *fat-6* and *fat-7* resulted in significant reduction in overall membrane maintenance. (D) The relative amount of synthesized FA is reduced with *fat-7* RNAi treatment (blue) compared to control RNAi (black). Numbers shown represent the mean of at least three experiments ± SEM. Statistical significance was determined by two-tailed unpaired t-tests (*p<0.05, **p<0.01, ***p<0.001).

We sought to identify regulators of membrane maintenance and to exclusively look for genes and pathways that impact day-to-day membrane maintenance, we initiated an adult-only RNAi approach at day 1 of adulthood after membranes have been fully formed. To validate our strategy, we targeted FA synthesis with RNAi of the *C*. *elegans* acetyl-CoA carboxylase gene, *pod-2*. After 48 hours of treatment, there is significant reduction in *de novo* synthesis as measured by ^13^C labeling, indicating the efficacy of the adult-only RNAi treatment (S4 Fig). Then, we examined the total amount of new fat provided to the membrane in *pod-*2 treated adults as *pod-2* depletions would limit the total amount of new fats available for the membrane. Indeed, we found a significant decrease in overall membrane maintenance in *pod-2* RNAi-treated animals (Fig 3C). We looked for altered dynamics in a very limited and specific subset of FAs by disrupting mmBCFAs through RNAi of *elo-5* and *elo-6* (Fig 3A). Both *elo-5* and *elo-6* RNAi resulted in a significant decrease in new monomethyl-branched chain FAs, C15iso and C17iso, but not any other FA species, indicating that the ^13^C method allows for specific monitoring of individual FA pools (Fig 3B).

### Stearoyl-CoA Desaturases Regulate the Replacement Rates of the Major Membrane Fatty Acids

As the types of FA species found in the membrane greatly impact its function, we targeted the enzymes of the FA elongation and desaturation (*elo/fat*) pathway (Fig 3A). For most enzymes, RNAi led to a reduction in the replacement rates of, at most, two FAs as seen in *elo-5* and *elo-6* RNAi. There is a more global impact on FA turnover with RNAi against the SCD genes, *fat-6* and *fat-7*, and the related palmitoyl-CoA desaturase, *fat-5*. Of importance, *fat-5*, *fat-6*, and *fat-7* can partially compensate for each other as single mutants are phenotypically wild-type and only the triple mutant is not viable [11]. Moreover, the sequence similarity of *fat-6* and *fat-7* results in the depletion of both gene products with either *fat-6* or *fat-7* RNAi treatment.

Both *fat-6* and *fat-7* RNAi led to a reduction in ^13^C-labeling, not only of its downstream products, but also in the majority of FAs examined, including the mmBCFAs (Fig 3B). The impact of SCD on fatty acid replacement can be quantified in *fat-6(tm331);fat-7(wa36)* double mutant animals (S5 Fig). The reduced dynamics seen in these mutants corroborates the finding from RNAi treatment and demonstrates this phenomenon is not RNAi-dependent. As the SCDs do not play a role in mmBCFA production, the impact of SCD RNAi on these mmBCFAs along with upstream species indicates a more wide-spread effect of SCDs on membrane maintenance. This finding is in contrast to the other *fat* and *elo* genes where the impacted FA species are confined to their downstream products.

### Stearoyl-CoA Desaturases Regulate Overall Membrane Maintenance

In order to compare the impact of SCDs on overall membrane maintenance, we calculated the total replacement rate of FAs in the membrane as done with the earlier comparisons of PL and NL populations. RNAi of *fat-5*, *fat-6* and *fat-7* resulted in a significant reduction in overall membrane maintenance while the other FA elongases (*elo-1*, *elo-2*, *elo-5*, *elo-6)* and desaturases (*fat-1*, *fat-2*, *fat-3*, *fat-4*) did not have a significant impact on overall membrane replacement (Fig 3C). Taken together, our analysis of membrane maintenance demonstrates that the *SCD*s play a specific role, not only in determining the types of fats that are available for PL synthesis, but also in regulating the rates of their incorporation.

Next, we explored how *fat-7* RNAi results in the downregulation of membrane maintenance. Because compromised *SCD* activity reduces *de novo* FA synthesis in other systems, we quantified *de novo* FA synthesis in *fat-6* and *fat-7* RNAi treated animals [28]. Indeed, we saw a reduction in lipogenesis with either RNAi treatment, suggesting that the compromised membrane replacement is, at least partially, through downregulation of *de novo* synthesis (Fig 3D). The decreased turnover of the cyclopropyl fatty acid, C17D, might suggest that food uptake is reduced, as these fatty acids are exclusively derived from the bacterial diet; however, we do not see a reduction in feeding as measured by pharyngeal pumping rate with *fat-7* treatment (see S6 Fig). Furthermore, there is not a significant change in the NL:PL ratio in the *fat-7* RNAi animals, indicating that fat stores are intact and not being consumed (see S6 Fig). Thus, decreased C17D replacement represents a decreased flux of new material to the PL and not reduced availability of dietary fat. The reduced membrane maintenance observed with *pod-2* RNAi further supports a role for lipogenesis and not food uptake in generating sufficient FA pools for membrane maintenance.

### A Novel ^15^N-Incorporation Assay Measures Intact Phospholipid Turnover

FAs are found in the membrane attached to a phospho-glycerol backbone, and, although ^13^C labeling revealed the continual rejuvenation of the FAs in the membrane, the dissociation of the FAs from the polar headgroup in our sample preparation for GC-MS prevented any insight into intact PL dynamics. An understanding of PL dynamics is vital, as the fatty acids are nearly always tethered to a head group, and, along with the movement of FAs in and out of the membrane, PL populations can also be consumed by cellular processes, requiring the phospholipids themselves to be replenished. Therefore, we developed a strategy to interrogate dynamics of individual, intact PLs using high-performance liquid chromatography (HPLC) and tandem mass spectrometry (MS/MS) based on recent work [29]. Our approach detected individual PLs and quantified their abundance with the Lipid Data Analyzer (LDA) software by considering exact mass, natural isotope distribution and retention time [30].

**Fig 4:**
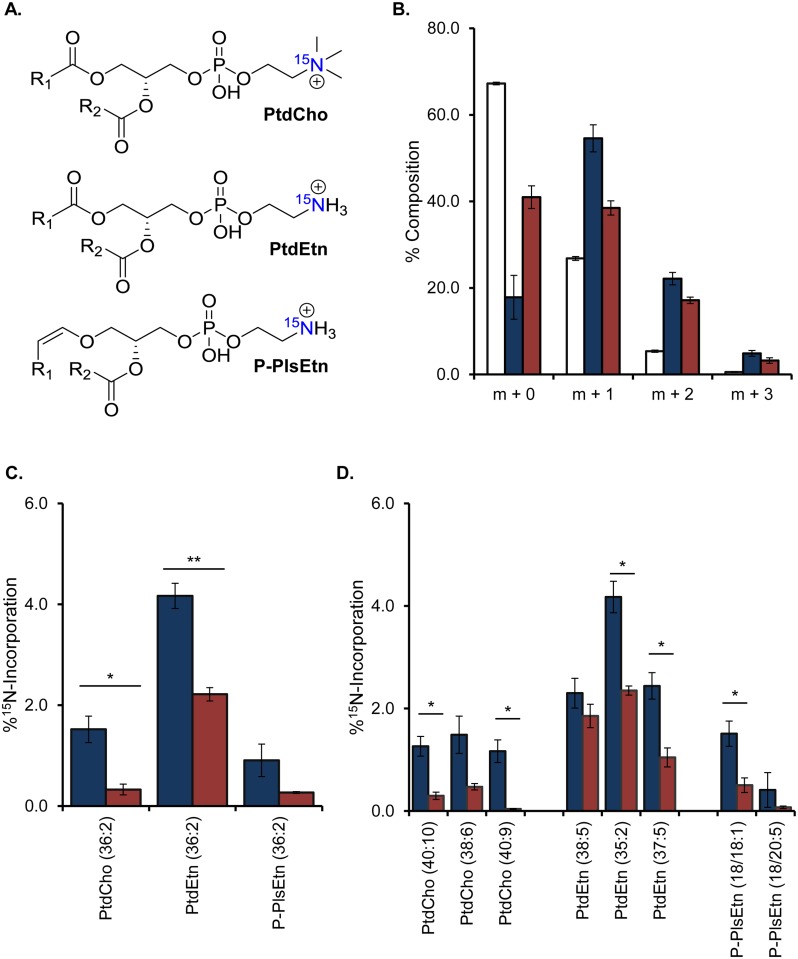
^15^N-Tracers and LC-MS/MS Measure PL Dynamics in Wild-Type and *fat-7* RNAi Animals. (A) Representation of the major PLs in the nematode: phosphatidylethanolamine (PtdEtn), phosphatidylcholine (PtdCho), and plasmenylethanolamine (P-PlsEtn) where R_1_ and R_2_ represent aliphatic carbon chains. The single nitrogen is highlighted in blue. (B) LC-MS/MS showed successful incorporation of ^15^N into the headgroups of PtdEtn (36:2) in labeled (blue) compared to unlabeled (white) control animals. Incorporation of ^15^N was compromised in this representative example for animals treated with *fat-7* RNAi (red). (C) The enrichment of ^15^N seen in each PL was modeled by % ^15^N-labeled PL per hour. The incorporation of ^15^N was significantly lower in *fat-7* RNAi treated animals (red) compared to control (blue). (D) The amount of new FAs for the most abundant species from each PL class is shown for control (blue) and *fat-7* RNAi treatment (red) as well. Data shown represent SEM and a minimum of n of 3. Significance was defined by two-tailed unpaired t-tests (*p<0.05, **p<0.01).

In order to characterize the membrane landscape, we tracked the most abundant PL classes in the nematode, phosphatidylcholine (PtdCho) and phosphatidylethanolamine (PtdEtn) which together comprise more that 85% of the membrane [31]. Further interrogation of the species with phosphoethanolamine headgroups revealed PtdEtn species with standard ester linkages and a unique subclass containing vinyl ether linkages called plasmenylethanolamines (P-PlsEtn) (Fig 4A). There are also alkyl derivatives called plasmanylethanolamines present, but they were not addressed in this study. Additionally, we looked for but did not find any significant abundance of plasmenylcholines or plasmanylcholines. In total, we detected a minimum of 230 distinct PL molecules when considering species as a summation of the two constituent *sn-1* and *sn-2* chains (see S2 Dataset). For instance, a PtdEtn 36:2 would define a phosphatidylethanolamine with 36 carbons and 2 total double bonds combined in its two FAs. Therefore, 36:2 might represent a molecule with two C18:1 chains or a C18:0 and a C18:2, demonstrating that our detection is an underestimate of membrane diversity.

This tremendous diversity, combined with the relatively small differences in molecular weight confounds the analysis if multiple stable isotopes are incorporated. Therefore, in order to measure intact PL turnover, we fed animals ^15^N-*E*. *coli* for 18 hours to incorporate a stable isotope at a single position in the polar headgroup (Fig 4A). We found that significant ^15^N accumulated in each population as shown in PtdEtn 36:2 (Fig 4B). Due to the sheer abundance of unique species, we focused our analysis on PtdCho and PtdEtn and P-PlsEtn species with the same tail combinations. The replacement of headgroups for PtdEtn 36:2 was 4.17 ± 0.25% each hour and almost 3 times that of either the PtdCho 36:2 or P-PlsEtn 36:2 (Fig 4C). Next, we examined the most abundant species for each lipid class, and, in doing so, a consistent trend emerged where the PtdCho population had slower dynamics than the PtdEtn population in all comparisons (Fig 4D). The faster dynamics were specific to PtdEtn with an average replacement rate of 3.7% per hour, with ether-linked P-PlsEtn (0.9%/hour) being replaced at rates more similar to PtdCho (1.4%/hour). Again, the unique dynamics of each PL population supported the need for individual assessment of populations to map overall membrane dynamics and to establish a methodology for understanding the regulation of PL turnover.

### Stearoyl-CoA Desaturases Regulate Phospholipid Turnover

Because SCD depletion slowed FA turnover in the membrane, we tested SCDs for a role in regulating PL maintenance. ^15^N accumulation in all PL species was severely compromised in nematodes treated with *fat-7* RNAi (Fig 4C and 4D). The replacement of most measured PL species was significantly compromised with an average fold-change of 0.36 when *fat-7* was targeted. The only PL without a significant reduction in turnover was PtdEtn 38:5 (20:5/18:0) with C20:5n3 and C18:0 tails. We hypothesize that PtdEtn 38:5 may be selectively maintained as a sink for the excess saturated C18 that accumulates in *fat-7* RNAi treatment. In summary, stable isotope tracers revealed a novel role for SCDs in influencing the rates of PL maintenance and, therefore, in providing the membrane with new resources for its maintenance.

### Describing the Impact of Stearoyl-CoA Desaturases on Phospholipid Remodeling

**Fig 5:**
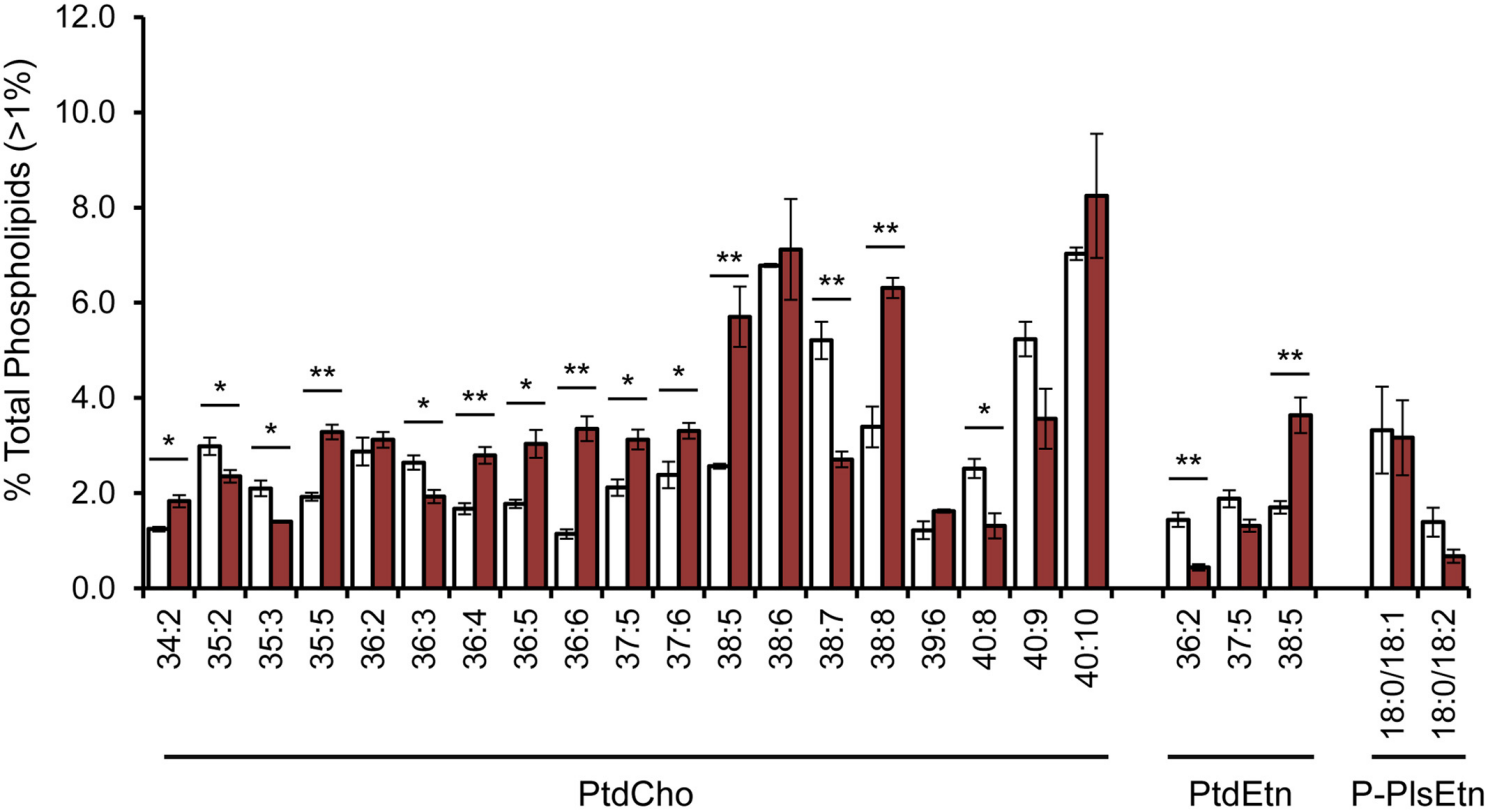
LC-MS/MS Analysis of PL Composition in *fat-7* RNAi Treated Animals. The major PL species (>2%) in control animals are represented by X:Y (where X is the number of carbons in both FAs tails and Y indicates the total double bonds). For a complete list of species, see source data (S2 Dataset). For P-PlsEtn, the LC-MS/MS detection allowed for both chains to be accurately reported. Control RNAi is represented with white bars, and *fat-7* RNAi is shown in red. The fatty acid tails for each species can be qualitatively assessed (see S7 Fig). Significant changes as are indicated by * (p<0.05) and ** (p<0.01) determined by two-tailed unpaired t-tests. Numbers shown represent the mean of at least three experiments ± SEM.

Because the replacement of both FA tails and PLs is compromised with *fat-7* RNAi, we used LC-MS/MS to determine how the composition of the membrane changes with decreased membrane maintenance. In *fat-7* treated animals, there were significant changes in the makeup of the membrane particularly in the PtdCho population (Fig 5). Although our methodology only allows for semi-quantitative determination of the FA tails found within each molecule, it was clear that the PtdCho molecules reduced in *SCD* RNAi-treated animals contained C18 and C20 PUFA species that are reflective of SCD activity (S7 Fig). For instance, the most reduced species was PtdCho 38:7 (20:5/18:2), suggesting alterations in the membrane due to reduced availability of PUFAs. There was an increase in species containing C20:5n3 fatty acids including PtdCho 38:5, PtdEtn 38:5, and PtdCho 40:10 that indicates an increase in this population. We cannot exclude the possibility that normalization artificially increases the apparent abundance of this species; however, we believe this apparent increase may indicate that any available polyunsaturated fatty acid is funneled specifically to maintain or even upregulate C20:5n3 production.

To compensate for altered FA supply, there was evidence supporting active PL remodeling with *fat-7* RNAi treatment as seen with the increase in PtdCho 38:8 which contains a mixture of PtdCho 38:8 (20:5/18:3) and PtdCho 38:8 (20:4/18:4). Both C18:3 and C18:4 are not highly abundant in the wild-type membrane, and the presence of these lipids suggests an adaptation of FA synthesis pathways to compensate for reduction in the canonical PUFAs that are found in the nematode. Similar alterations have been observed in fatty acid analysis of *fat-6;fat-7* mutants with the accumulation atypical fatty acid species [11].

Next, we examined the abundance of PtdEtn 38:5 (20:5/18:0) as it is the only molecule we found that is not impacted by *fat-7* RNAi treatment (Fig 4D). The PtdEtn 38:5 was upregulated with more than double the PtdEtn 38:5 found in *fat-7* animals (Fig 5). This significant upregulation supports a selective resorting of acyl chains to pair the abundant C18:0 FAs with highly unsaturated species. This pairing may offset the impact of the increased saturated fat on the biophysical properties of the membrane by maximizing the total number of double bonds in PLs containing C18:0. In support of this model, there is an even greater accumulation of PtdCho 38:5 (20:5/18:0) representing another favorable pairing for the excess saturated FAs (Fig 5). Taken together, SCDs play a significant role not only in providing the proper FA species for PL synthesis but also in instructing the pairing of unfavorable acyl chains to mitigate changes in overall membrane structure.

## Discussion

**Fig 6:**
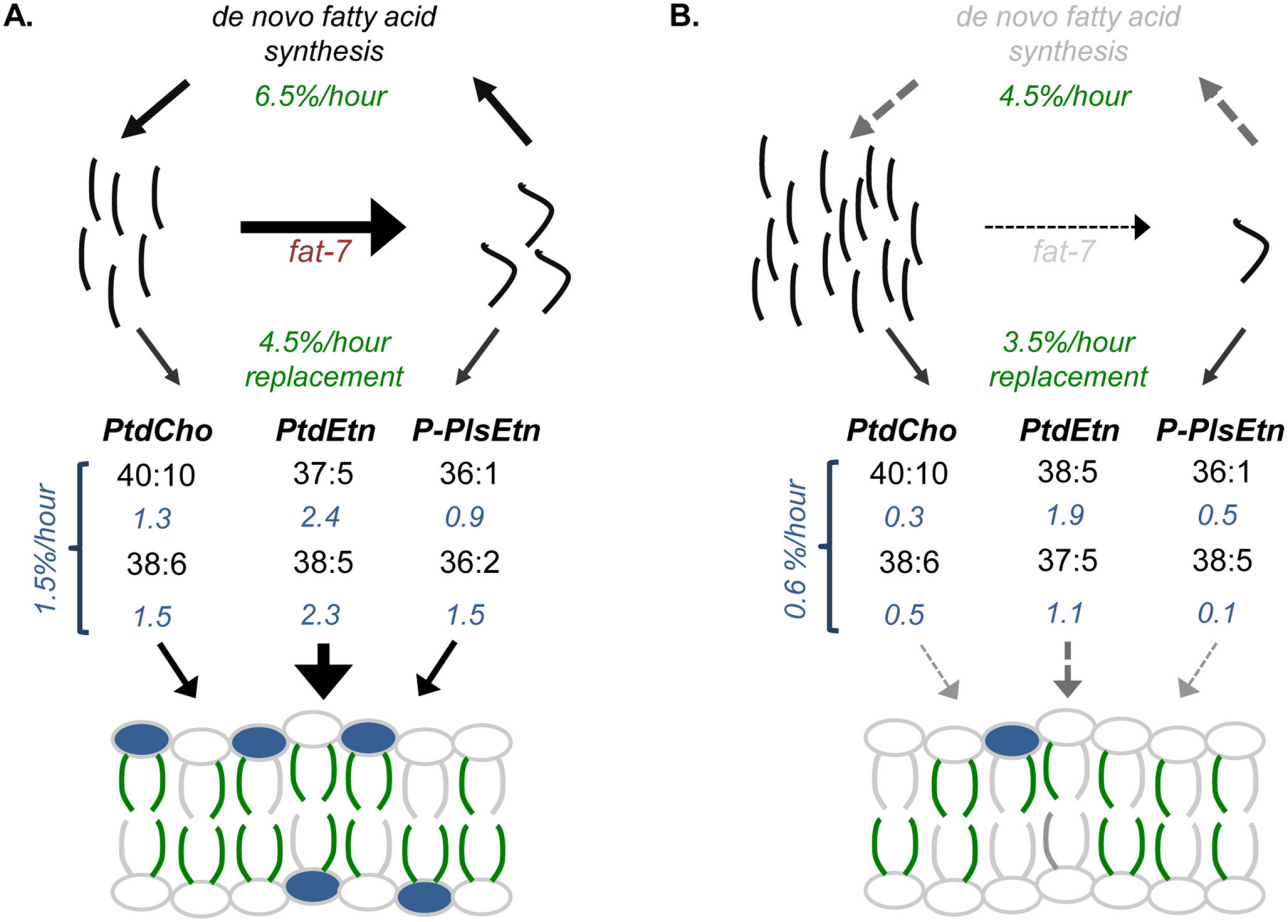
Modeling the Impact of *SCD* Depletion on Membrane Dynamics. (A) ^13^C-tracers allow for the quantification of *de novo* synthesis as well as incorporation of FAs into PLs (measurements from ^13^C-tracers shown in green). Our implementation of ^15^N to measure PL headgroup turnover defines the rates that the two most abundant species within each major PL class are replaced (shown in blue); for example, 40:10 and 38:6 are the largest contributors to the PtdCho pool and are replaced at a rate of 1.3% new PL/hour and 1.5% new PL/hour respectively. A weighted average of the PL replacement calculated for the PL species reported here predicts that 1.5% of the total PLs are replaced each hour. We combined the PL replacement rates with those calculated for FA tails where we found that 4.5% of measured fatty acid tails are new each hour. The quantification after 24 hours revealed a stable population of membrane lipid (see S3 Fig); thus, we reduced our average fatty acid predictions by 30% to account for that population. Taken together, we use the ^13^C and ^15^N-tracers to define how the individual lipid components (FA tails and polar headgroups) are replaced over time, and predict that at least 60% of the membrane is new each day when considering each fatty acid tail and headgroup as individual entities. (B) Upon *fat-7* RNAi treatment, there is a buildup of C18:0 and a depletion of C18:1n9, which in turn reduces the activation of *de novo* FA synthesis. Subsequently, FAs provided to PL synthesis and ultimately provided to the membrane, are severely compromised.

Membranes are not static structures, and, therefore, it is important to not only understand their composition, but also how they are maintained over time. We have utilized recent advances in mass spectrometry and lipidomic analysis to define, in detail, the membrane composition of *C*. *elegans*; however, these approaches alone do not consider the constant flux of the lipid bilayer. To address the question of membrane dynamics, we developed mass spectrometry-based tools to monitor the general turnover of FAs and intact PLs using ^13^C- and ^15^N-isotope labeling strategies, respectively. Importantly, the use of stable isotope-enriched diets does not only give us one measure of membrane dynamics, but this labeling scheme also allows for the simultaneous monitoring of many individual populations. By combining multiple methods of mass spectrometry with stable isotope feeding paradigms, we can report the most thorough characterization of phospholipid dynamics to date (Fig 6).

The turnover of membrane fatty acids is very rapid, and, although there has been a great focus on the role of fat stores in health and longevity, we find that more dietary resources are funneled into PL maintenance, highlighting a demand for maintaining the membrane over generating additional fat stores. The rapid lipid replenishment occurs after the main reproductive period of the animal, and, thus, the continual lipid turnover seems to demonstrate a futile cycle; however, we hypothesize that without new dietary resources the membrane composition would degrade over time. In fact, it is likely that altered provision of resources to the membrane would have serious consequences on its structure and could be a crucial component of the altered membrane composition observed in many diseases including cancers. The lack of a model system has occluded an understanding of how the membrane impacts disease progression and treatment. The establishment of a methodology to measure membrane dynamics in the nematode will ultimately allow for the ability to probe the role of PL maintenance in health and disease.

Using the rates of ^13^C incorporation and FA abundance, we predicted that the entire phospholipid population would be replaced within 24 hours. In fact, a longer labeling period demonstrated that the majority of the membrane (>70%) is new within a day. There are certain fatty acids where the amount of turnover over the 24-hour period was less than predicted by the hourly rate determined in the 6-hour labeling period. For those species, we predict that the location of the fatty acids in individual membranes or domains may limit the turnover in that population (S3 Fig). We anticipate that expanding our techniques to sub-cellular membrane compartments will reveal insights into localized membrane maintenance within the cell. Regardless, measurements from total phospholipid clearly demonstrate that the majority of membranes will have new fatty acids within a day. Likewise, tissue-specific regulation will almost certainly play a role in the preservation of membranes. In fact, it has been well-established, in *C*. *elegans*, that the nervous system can impact fat metabolism in other tissues [32, 33]. As our methods have revealed novel insights of membranes from whole animals, their application to tissues, individual organelle membranes and domains will be informative, and advances in mass spectrometry may allow such studies in the near future.

Here, we conducted the first *in vivo* measurements of the phospho-headgroups using ^15^N stable isotopes and, in doing so, found that an average of ~2% of the PL pools were replaced each hour. Not only do these independent measurements of membrane dynamics corroborate the rates of replacement observed in the FA populations, but they also allow for comparisons between the flux of membrane tails and headgroups. In the event that an entire PL moiety was recycled, each quantified PL turnover event would represent the replacement of two FA tails; therefore, the average turnover determined with ^15^N needs to be doubled to be consistent with measurements of the ^13^C in the tails. Taking this into account, the corrected turnover of the headgroups and the FA tails are roughly equivalent, implicating PL recycling as the primary mechanism of membrane maintenance in young adults. Other important considerations in membrane maintenance are the roles that acyltransferases and phospholipases play in membrane homeostasis. Acyltransferases may be particularly important in membrane adaptation, as studies have highlighted their role in remodeling fatty acid composition in response to different environments [17, 34]. Future studies targeting PL pathways, such as phospholipases and acyltransferases, are needed to expand the understanding of the maintenance network under both basal and stress conditions.

Because *C*. *elegans* is amenable to genetic studies, RNAi methods were used to validate our stable isotope approaches; however, targeting the *elo/fat* pathway by RNAi also led to the characterization of the SCDs and their critical impact on the rates of membrane maintenance. SCDs are essential for the generation of the PUFAs in larval animals, and, therefore, we used a short-term, adult-only RNAi approach that allows us to examine the immediate response to gene disruption, bypassing the role of SCDs in development. Not only will this RNAi approach be useful for the identification of genes that impact adult lipid metabolism, but, unlike the total depletion of gene products seen in null mutants, short-term RNAi may more accurately model many disease states. Moreover, the trends defined with RNAi are consistent with mutants in the *SCD* genes (S5 Fig). Because the membrane turns over so quickly, we can see changes in membrane composition, namely the depletion of most C18 and C20 PUFAs, after only 48 hours of RNAi treatment. The ability to interrogate the role of SCDs in the regulation of membrane replacement independent of any developmental roles allowed us to specifically assess the function of these genes in membrane maintenance.

The fast turnover of the PLs demonstrates the need for a constant infusion of new material to maintain proper membrane composition. When we targeted the ability of the nematode to provide sufficient material to the membrane with *fat-7* RNAi, there were a number of significant changes in membrane structure, even with a relatively short RNAi treatment window. Using LC-MS/MS analysis, we found an increase in atypical FA species, particularly in the PtdCho population, indicating altered FA processing to alleviate FA composition stress similar to what has been found in the *fat-6;fat-7* double mutants [11]. Additionally, there was a significant increase of PL species containing C18:0 saturated fat, and we found that the C18:0 is frequently paired with a highly polyunsaturated FA chain. We predict that the upregulation of this pairing would minimize the impact of saturated FA accumulation in the membrane and consume the abundant C18:0 chains with minimal impact on the membrane’s biophysical properties. Taken together, this work indicates that changes in *SCD* expression such as those seen in cancers and metabolic disorders would have important consequences to membrane function. The role of PL maintenance and composition in disease is not fully understood; however, it certainly warrants further study particularly as SCD is an active drug target, and its dysregulation has been directly implicated in the onset of cancer [35, 36].

Although general measurements of membrane dynamics have been reported for other systems using exogenously provided tracers, these studies have been restricted in their scope and have not been conducted in organisms where the regulatory pathways can be thoroughly probed. The affordability and relatively high-throughput nature of the stable isotope-labeling paradigm in the nematode establishes the framework for the future definition of genes and conditions that impact membrane maintenance. The combination of GC-MS and LC-MS/MS has allowed us to probe the major lipid species within the membrane in great detail. The tools described here can be applied to most FA and PL species and will expand the capabilities of lipidomics. Application of these methods to other systems will generate novel insights into how lipids change under various conditions and how that response is orchestrated. In short, we present a thorough quantification of wild-type membrane dynamics, identify critical new consequences of SCD perturbation in the membrane, and introduce tools that will serve as a platform for further exploration of lipid metabolism.

## Materials and Methods

### Nematode Strains and Growth Conditions

Unless noted, experiments used the temperature-sensitive sterile strain, CF512 (*fer-15(b26);fem-1(hc17))* maintained on *E*. *coli* (OP50). Embryos were harvested by bleaching gravid adults and hatching the resulting eggs in M9 for 20–24 hours at 20°C. Synchronized L1s were grown at 25°C at a density of 5,000 animals per 10 cm HG plate to induce sterility. Strains were obtained from the *Caenorhabditis* Genetics Center (CGC, Minneapolis, MN).

### Adult-Only RNAi Feeding Strategy

Starter cultures of HT115 bacteria transformed with control RNAi (empty *L4440* vector) or RNAi of interest from the Ahringer library were used to inoculate large-scale cultures (approximately 100mL LB) containing 50 μg/mL carbenicillin and 15 μg/mL tetracycline. Each culture was grown for 16 hours at 37°C, harvested by centrifugation and plated at a density of 0.15g per 10 cm NGM-CI plate. Plates were incubated in 25°C for 48 hours and then young adult worms (44 hours after plating) were transferred at a density of 2,500 worms per plate. After 48 hours, day 3 adults were harvested, washed with M9 and moved onto isotope labeling plates as described below.

### Stable Isotope Feeding Approach

^13^C feeding plates were prepared as previously described [9]. Briefly, OP50 colonies were separately inoculated in ^12^C-LB or ^13^C-Isogro (Sigma) media for 16 hours at 37°C, harvested and resuspended in M9 at a concentration of 0.15g/mL. Each 10 cm agarose plate was seeded with 1mL of 1:1 mixture of ^12^C:^13^C, dried for 15 minutes and 5,000 synchronized day 3 adults were transferred to each isotope feeding plate for 6 hours at 25°C. Nematodes were harvested, washed, frozen in a dry ice/ethanol bath and stored in -80°C. For each replicate, a bacteria-only plate was collected to determine the exact ratio of ^12^C:^13^C (S1 Fig). The same protocols were used for ^15^N except animals were fed the 100% ^15^N-OP50 for 18 hours to ensure sufficient enrichment.

### Lipid Extraction and GC-MS Data Analysis

Total lipid was extracted, and PL and NL populations were prepared for GC-MS as described [9, 37, 38]. Briefly, internal lipid standards, 1,2-diundecanoyl-*sn*-glycero-3-phosphocholine (Avanti Polar Lipids) and tritridecanoin (Nu-Chek Prep), were added to each sample, and total worm fat was extracted with chloroform:methanol (2:1) for 1.5 hours at room temperature. Dried total lipids in 1mL of chloroform were loaded onto HyperSep Silica SPE columns (100mg capacity, Thermo Scientific), and NLs, glycosphingolipids, and PLs were collected. Purified PLs and NLs were dried and resuspended in 1mL of 2.5% H_2_SO_4_ in methanol, then incubated for 1 hour at 80°C to create fatty acid methyl esters (FAMEs) to run on GC-MS (Agilent 5975GC, 6920MS).

Relative FA composition was determined by comparing the integrated area for each FA peak over the total integrated area for all major fatty acids. For most species, the mass spectra across the FA peak were averaged to determine the abundance of each isotopomer. In select species, presence of other molecules at the tails of the peak required the exclusion of contaminated scans (see S8 Fig). For C18 FAs, % new FAs was calculated from the following isotopomers after subtraction of the natural abundance of ^13^C (1.1%) in the environment by:

[(∑m+1,…,m+13)+(∑m+0,…,m+18)]*100 (1)

where CF is a correction factor for any deviation from a 1:1 ratio of ^12^C- to ^13^C-OP50. This value accounts for ^13^C incorporation events (m+1,…,m+13 isotopomers) such as via *de novo* FA synthesis. ^13^C-FAs are also derived from intact dietary FA, and the elongation of fully unlabeled and labeled C14:0 and C16:0. Together, these sources can be accounted for by multiplying the fully labeled and elongated species (represented by m+14,…, m+18) by CF to account for the incorporation of unlabeled dietary FA. Relative *de novo* synthesis was quantified as reported [9]. Briefly, acetyl-CoA molecules used to build fatty acids incorporate stable isotopes and can have 0 to 2 stable isotopes. This distribution of ^12^C-^12^C, ^12^C-^13^C, and ^13^C-^13^C units can be directly measured in the nematode by monitoring the extension of a fully labeled C16:0 into a C18:0 which does not occur in the bacterial diet. The distribution can then be applied to assess the amount of fatty acids derived from synthesis. All fatty acid species were analyzed similarly except C20:4 and C20:5 which fragment extensively.

### LC-MS/MS Conditions and Detection

Total lipid samples for whole worm LC-MS/MS analysis were generated as described above. For a typical 10,000 animal sample, 200 μL of the LC-MS/MS dilution buffer, acetonitrile/2-propanol/water (65:30:5 v/v/v), was used to dissolve the lipids prior to LC-MS/MS injection [29]. Next, 10 μL of the resuspended lipids were then injected onto the LC-MS/MS for negative ion scanning mode.

Lipid samples were separated using a C18 Hypersil Gold 2.1 x 50 mm, 1.9 μm column (25002–052130; Thermo Scientific) equipped with a 2.1 mm ID, 5 μm Drop-In guard cartridge (25005–012101; Thermo Scientific). The column was connected to an Accela 600 quaternary pump system, a Thermo Scientific Autosampler, and an LTQ-Orbitrap mass spectrometer (Thermo Scientific). The HPLC solvent selection and schedule were the same as reported [29]. The following parameters were used: column oven temperature at 50°C, autosampler tray at 7°C, the flow rate at 300 μL/min, scan range between *m/z* 300–1200, the capillary temperature at 250°C, the sheath gas flow at 25 units, the auxiliary gas flow at 15 units, and the source voltage was 3.5 kV. The machine was tuned in both positive ion and negative ion mode using a 10 μM (7.9 ng/μL) sample of 1,2-distearoyl-*sn*-glycero-3-phosphocholine (Avanti Polar Lipids) dissolved in a mixture of mobile phase A and B (50:50 v/v). For MS1 profiling, scans were run at a resolution of 60k. MS2 analyses were performed using 6 scan events, where the top five ions were chosen from an initial MS1 scan. For fragmentation, a normalized collision energy of 35 was used. An exclusion duration of 15 sec was used with an exclusion mass width of low = 0.55 and high = 1.55. MS1 spectra were collected in profile mode and MS2 collected in centroid mode. Charge states 2–4 and any unassigned states were rejected.

### PL Composition and ^15^N Analysis

Analysis was performed using the LDA software (Version 1.6.2.5) [30]. Full profile MS1 scans in negative ion mode were used for quantitation. Within LDA, the OrbiTrap XL settings were used with a modified profile Peak Acceptance Range = 0.005. The negative ion mode MS1 RAW files were analyzed using a 0% relative peak cut-off value. LDA exact mass lists were generated for PtdCho, PtdEtn, P-PlsEtn, and P-PlsCho using the following molecular ranges: for PtdCho and PtdEtn, molecular species with combined carbon-lengths of 24–48 and 0–12 degrees of unsaturation were quantitated. For P-PlsCho and P-PlsEtn, the alkyl- and alkenyl-ether tails of carbon-length: degrees of unsaturation (15:0–20:0) were evaluated with *sn-2* tails of carbon-length 8, 10, 12–24, with degrees of unsaturation ranging from 0–12. MS2 spectra helped to validate LDA species identifications, which, when possible, could also be compared with predicted fragmentation patterns using the LIPID MAPS product ion prediction tools: (www.lipidmaps.org). No significant amount of P-PlsCho was found.

^15^N-isotope incorporation rates were determined by manually measuring the isotope peak intensities using the Thermo Qual Browser Software in Xcalibur version 2.2 (Thermo Scientific). The prominent isotopic peaks for the selected species and their intensities were quantified for the spectra. A 100% ^14^N sample was used to determine the natural isotope abundance, which can be subtracted from the ^15^N-labeled animals to define the incorporation of the exogenous ^15^N. The 98% purity of the ^15^N-ISOGRO powder was also accounted for before the quantification of MPE by:

[(∑m+1,…,m+4)/(∑m+0,…,m+4)]*100 (2)

## Supporting Information

S1 Dataset Fatty Acid Composition With Various RNAi Treatments. (XLSX)

S2 Dataset Complete List of Phospholipids Detected for PtdCho, PtdEtn, P-PlsEtn for Control and fat-7 RNAi. (XLSX)

S1 Fig Stable Isotope Labeling of *E*. *coli* Diet. The isotopomer distribution from the ^12^C:^13^C bacterial mixture demonstrates that there is no mixing of individual carbons between the unlabeled and stable isotope-labeled bacteria as there are no significant isotopomers between the molecular weights (MW) of 299 and 311. The exact ratio of the ^12^C:^13^C diet is determined for each experiment by comparing the abundance of the exclusively ^13^C peaks (MW: 312–314) to the ^12^C peaks (MW: 296–298). Data from 18 experiments with SEM shown.
(PDF)

S2 Fig Comparative Analysis Between *fer-15;fem-1* and WT Adults. In order to explore how the sterile adults (*fer-15;fem-1*, green) relate to wild-type (N2, gray) adults, we compared the rates of new fatty acid incorporation at two time points, day 1 (D1) and day 3 (D3) of adulthood. At D1, there is no significant difference in the dynamics of the fatty acid populations between the two groups, indicating that *fer-15;fem-1* is a viable model for fat metabolism in early adults. By D3, the *fer-15;fem-1* animals had a significant decrease in fatty acid turnover. This decrease is not nearly as dramatic in the WT population; however, we believe that the numbers are artificially elevated by the presence of contaminating larval animals that have higher rates of turnover.
(PDF)

S3 Fig Extended ^13^C-Labeling Period Reveals the Majority of Membrane Fatty Acids are Replaced After 24 Hours. A population of day 3 adult nematodes was fed a diet of ^12^C:^13^C as in Fig 2A but for 24 hours instead of 6 hours. The analysis from the 6-hour feeding period predicted that each fatty acid population would be replaced entirely within this 24-hour period (white bars). The dashed line marks 100% replacement indicating the maximum replacement that can be measured. The amount of new fatty acid/hour calculated from the longer period (green) shows that there is very significant turnover after 24 hours from 56.6 ± 1.0% in C18:2n6 to 80.8 ± 1.3% in C16.1n7, supporting the rapid dynamics defined by the 6-hour labeling. In some cases like C18:0 and C18:2n6, the predictions align well with the experimental data. For the other species, there is separation between measured and predicted values with the largest difference seen in C16:0. We predict that the disparity between the numbers indicates the presence of a stable population of lipids that have slower dynamics, perhaps due to their subcellular location. SEM is shown; n = 4.
(PDF)

S4 Fig Reduced Fatty Acid Synthesis in *pod-2* RNAi Treated Animals. The relative amount of synthesized fatty acid is reduced with *pod-2* (gray) RNAi treatment compared to control (L4440) RNAi (black). This significant decrease demonstrates the effectiveness of the short-term adult-only RNAi and confirms our ability to measure synthesis with a 6-hour labeling period. Numbers shown represent the mean ± SEM, n = 5. Statistical significance was defined by t-tests (**p<0.01, ***p<0.001).
(PDF)

S5 Fig *fat-6;fat-7* Double Mutants Verify the Role of SCDs in Fatty Acid Replacement. To corroborate the decreased fatty acid incorporation seen after *fat-7* RNAi treatment, we measured the amount of new fatty acids found in the phospholipid population of SCD mutant animals. Mutations in the *fat-6* or the *fat-7* desaturase have been shown to compensate for each other, and, because the RNAi against *fat-7* also targets *fat-6*, we analyzed *fat-6(tm331);fat-7(wa36)* animals. These animals have very limited progeny production, and, therefore, we used fertile animals with very minimal larval contamination. There is significant developmental delay in *fat-6;fat-7* nematodes, and there is not complete synchrony in the populations when assessed at day 3 of adulthood. Despite the technical challenges, the *fat-6;fat-7* animals (purple) show significantly reduced fatty acid turnover in phospholipids when compared to *fem-15;fer-1* control animals (black), similar to our observations with *fat-7* RNAi. Numbers shown represent the mean ± SEM, n = 6. Unpaired t-tests established significance (*p<0.05, **p<0.01, ***p<0.001).
(PDF)

S6 Fig Food Intake and Fat Storage are not Compromised with *fat-7* RNAi Treatment. (A) Pharyngeal pump rates were measured for *fat-5*, *fat-6*, and *fat-7* RNAi-treated animals to examine food intake. There was no significant change in food consumption in *fat-5* or *fat-7* RNAi-treated animals. There was a significant decrease in the pump rates of animals fed RNAi against *fat-6* which may contribute the greater impact on overall membrane maintenance (see Fig 3C). Minimums of 35 animals were counted over three distinct experiments. (B) There is no significant change in fat storage as measured by the ratio of neutral lipid (NL) to phospholipid (PL) with any RNAi treatment. *fat-6* RNAi-treated animals nearly met significance (p = 0.0506) which again highlights the more pronounced phenotype with this construct. Numbers shown represent the mean ± SEM, n = 3. Statistical significance was defined by t-tests (**p<0.01).
(PDF)

S7 Fig Assessment of Fatty Acid Tail Composition by LC-MS/MS. (A) Mass spectra can be used to identify the fatty acid tails associated with a given phospholipid population. Using the S2 scan from LC-MS/MS analysis, phosphatidylcholine (PtdCho) 38:7 shows the mass of two fragments (279.33, 301.25), indicating that the species consists of a C18:2 and a C20:5 fatty acid tail. (B) The MS2 scan of phosphatidylethanolamine (PtdEtn) 36:2 reveals two different species. Fragment masses 279.27, 281.33, and 283.40 correspond to fatty acid tails C18:2, C18:1, and C18:0. The MS2 scan shows that the PtdEtn 36:2 consists of one molecule with two C18:1 tails and another with C18:0 and C18:2 tails. (C) Table specifying fatty acid tail breakdown estimates for each listed PL species for both L4440 RNAi and *fat-7* RNAi.
(PDF)

S8 Fig Analysis of GC-MS Data in *fat -7* RNAi Treated Animals. (A) Scanning ion mode (SIM) profiles in control animals produced sufficient isotopomer abundance for both (i) C18:2n6 and (ii) C18:1n9 species. Therefore, we averaged the scans across the whole peak. (B) In contrast, the C18:2n6 SIM profile of *fat-7* RNAi treated animals had inseparable C18:3 contamination in the certain scans. In order to minimize the isotopomer bias in this species, we manually examined each scan and excluded a scan from the C18:2n6 when the height of m/z 292 is over 75% of the m/z 294. For C18:1n9, scans were similarly excluded when scan height of m/z 294 is under 75% of m/z 296.
(PDF)

S1 Table Fatty Acid Tail Composition of Day 3 *fem-1;fer-15* on RNAi. The relative abundance (%) of each fatty acid species of purified phospholipid (PL) and neutral lipid (NL) tails measured by gas chromatography. Data is presented as the average of 5 independent experiments ± SEM.
(PDF)
