# Supplementary material for: 13C- and 15N-Labeling Strategies Combined with Mass Spectrometry Comprehensively Quantify Phospholipid Dynamics in C. elegans
Source: PLoS One. 2015 Nov 3;10(11):e0141850. doi: 10.1371/journal.pone.0141850 (PMC4631354; doi:10.1371/journal.pone.0141850)
Supplement: S1 Fig — The isotopomer distribution from the 12C:13C bacterial mixture demonstrates that there is no mixing of individual carbons between the unlabeled and stable isotope-labeled bacteria as there are no significant isotopomers between the molecular weights (MW) of 299 and 311. The exact ratio of the 12C:13C diet is determined for each experiment by comparing the abundance of the exclusively 13C peaks (MW: 312–314) to the 12C peaks (MW: 296–298). Data from 18 experiments with SEM shown. (PDF) [file pone.0141850.s003.pdf]

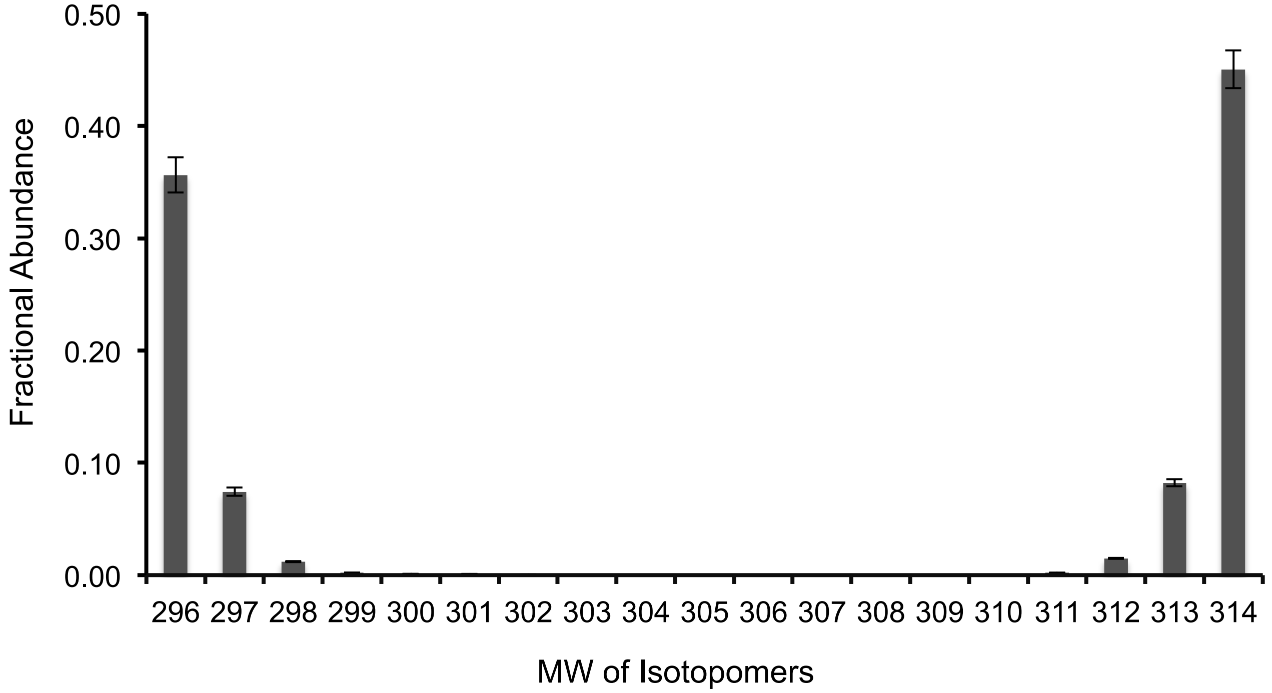

S1 Fig. Stable Isotope Labeling of *E. coli* Diet. The isotopomer distribution from the ^{12}C:^{13}C bacterial mixture demonstrates that there is no mixing of individual carbons between the unlabeled and stable isotope-labeled bacteria as there are no significant isotopomers between the molecular weights (MW) of 299 and 311. The exact ratio of the ^{12}C:^{13}C diet is determined for each experiment by comparing the abundance of the exclusively ^{13}C peaks (MW: 312-314) to the ^{12}C peaks (MW: 296-298). Data from 18 experiments with SEM shown.
