# Supplementary material for: 13C- and 15N-Labeling Strategies Combined with Mass Spectrometry Comprehensively Quantify Phospholipid Dynamics in C. elegans
Source: PLoS One. 2015 Nov 3;10(11):e0141850. doi: 10.1371/journal.pone.0141850 (PMC4631354; doi:10.1371/journal.pone.0141850)
Supplement: S2 Fig — In order to explore how the sterile adults (fer-15;fem-1, green) relate to wild-type (N2, gray) adults, we compared the rates of new fatty acid incorporation at two time points, day 1 (D1) and day 3 (D3) of adulthood. At D1, there is no significant difference in the dynamics of the fatty acid populations between the two groups, indicating that fer-15;fem-1 is a viable model for fat metabolism in early adults. By D3, the fer-15;fem-1 animals had a significant decrease in fatty acid turnover. This decrease is not nearly as dramatic in the WT population; however, we believe that the numbers are artificially elevated by the presence of contaminating larval animals that have higher rates of turnover. (PDF) [file pone.0141850.s004.pdf]

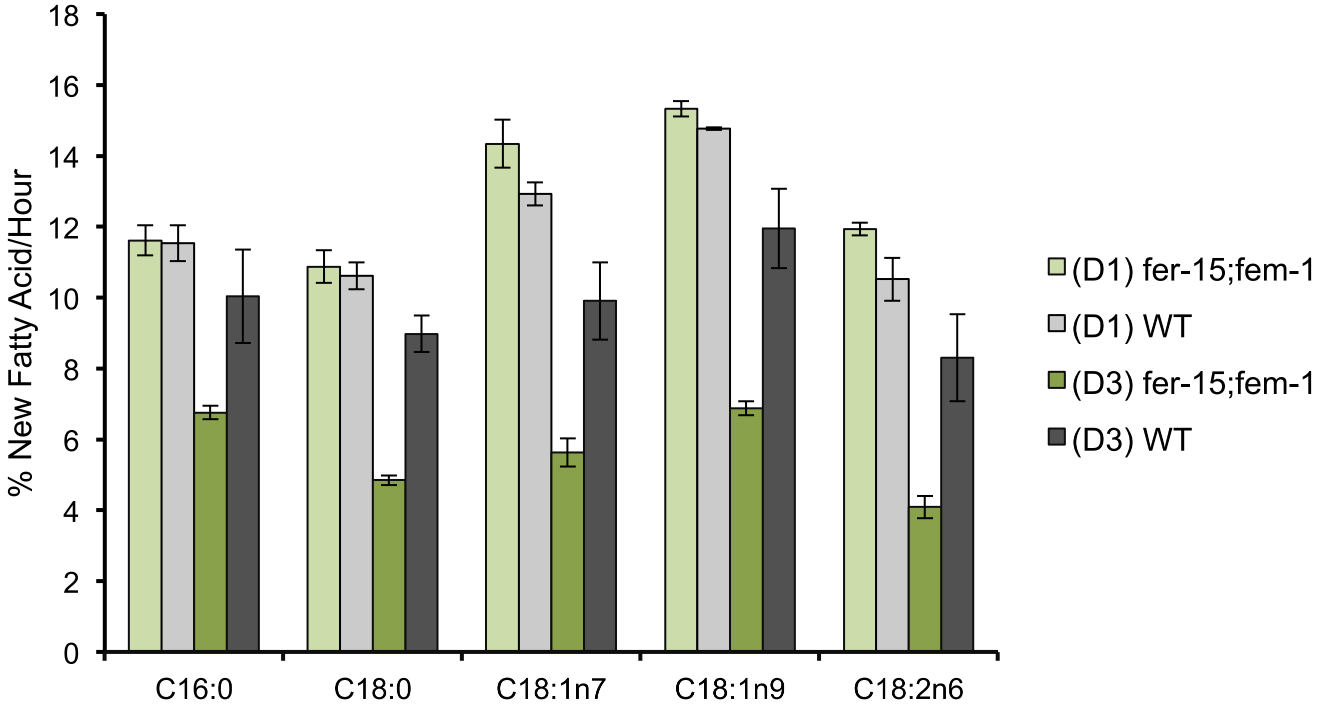

S2 Fig. Comparative Analysis Between *fer-15;fem-1* and WT Adults. In order to explore how the sterile adults (*fer-15;fem-1,* green) relate to wild-type (N2, gray) adults, we compared the rates of new fatty acid incorporation at two time points, day 1 (D1) and day 3 (D3) of adulthood. At D1, there is no significant difference in the dynamics of the fatty acid populations between the two groups, indicating that *fer-15;fem-1* is a viable model for fat metabolism in early adults. By D3, the *fer-15;fem-1* animals had a significant decrease in fatty acid turnover. This decrease is not nearly as dramatic in the WT population; however, we believe that the numbers are artificially elevated by the presence of contaminating larval animals that have higher rates of turnover.
