# Supplementary material for: 13C- and 15N-Labeling Strategies Combined with Mass Spectrometry Comprehensively Quantify Phospholipid Dynamics in C. elegans
Source: PLoS One. 2015 Nov 3;10(11):e0141850. doi: 10.1371/journal.pone.0141850 (PMC4631354; doi:10.1371/journal.pone.0141850)
Supplement: S3 Fig — A population of day 3 adult nematodes was fed a diet of 12C:13C as in Fig 2A but for 24 hours instead of 6 hours. The analysis from the 6-hour feeding period predicted that each fatty acid population would be replaced entirely within this 24-hour period (white bars). The dashed line marks 100% replacement indicating the maximum replacement that can be measured. The amount of new fatty acid/hour calculated from the longer period (green) shows that there is very significant turnover after 24 hours from 56.6 ± 1.0% in C18:2n6 to 80.8 ± 1.3% in C16.1n7, supporting the rapid dynamics defined by the 6-hour labeling. In some cases like C18:0 and C18:2n6, the predictions align well with the experimental data. For the other species, there is separation between measured and predicted values with the largest difference seen in C16:0. We predict that the disparity between the numbers indicates the presence of a stable population of lipids that have slower dynamics, perhaps due to their subcellular location. SEM is shown; n = 4. (PDF) [file pone.0141850.s005.pdf]

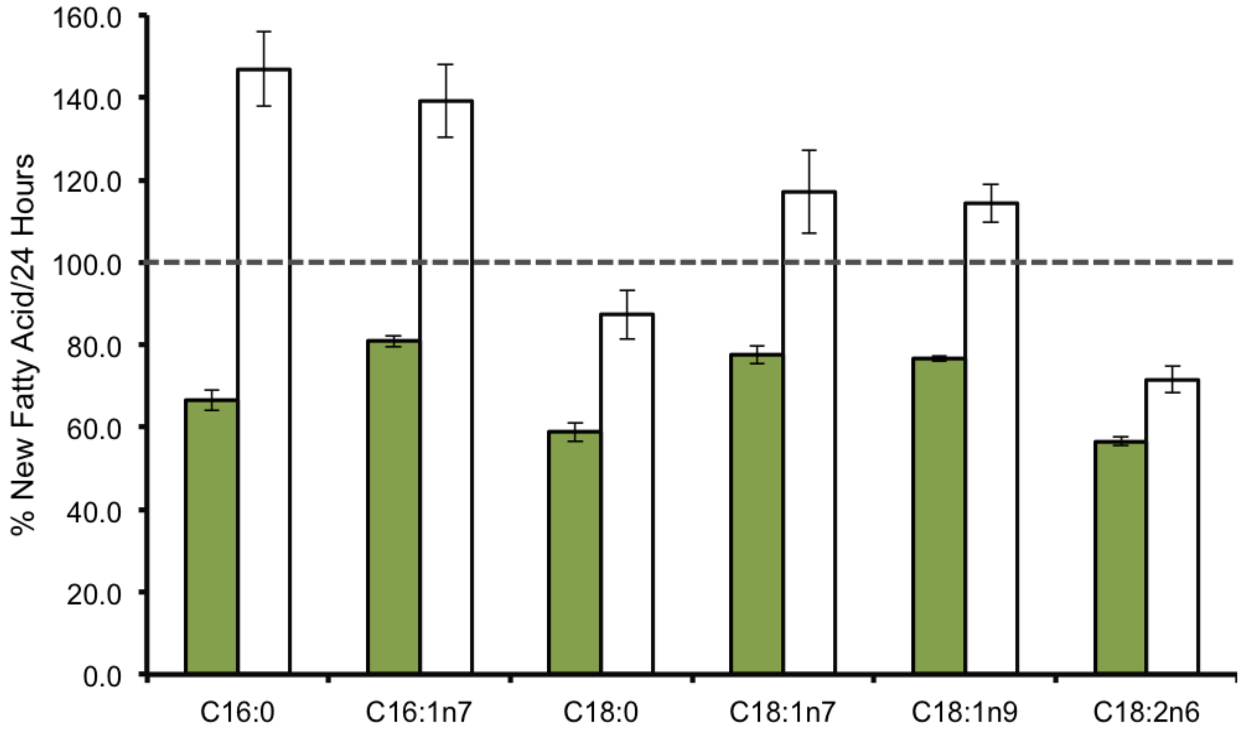

S3 Fig. Extended ^{13}C-Labeling Period Reveals the Majority of Membrane Fatty Acids are Replaced After 24 Hours. A population of day 3 adult nematodes was fed a diet of ^{12}C:^{13}C as in Fig 2A but for 24 hours instead of 6 hours. The analysis from the 6-hour feeding period predicted that each fatty acid population would be replaced entirely within this 24-hour period (white bars). The dashed line marks 100% replacement indicating the maximum replacement that can be measured. The amount of new fatty acid/hour calculated from the longer period (green) shows that there is very significant turnover after 24 hours from 56.6 ± 1.0% in C18:2n6 to 80.8 ± 1.3% in C16.1n7, supporting the rapid dynamics defined by the 6-hour labeling. In some cases like C18:0 and C18:2n6, the predictions align well with the experimental data. For the other species, there is separation between measured and predicted values with the largest difference seen in C16:0. We predict that the disparity between the numbers indicates the presence of a stable population of lipids that have slower dynamics, perhaps due to their subcellular location. SEM is shown; n=4.
