# Supplementary material for: 13C- and 15N-Labeling Strategies Combined with Mass Spectrometry Comprehensively Quantify Phospholipid Dynamics in C. elegans
Source: PLoS One. 2015 Nov 3;10(11):e0141850. doi: 10.1371/journal.pone.0141850 (PMC4631354; doi:10.1371/journal.pone.0141850)
Supplement: S4 Fig — The relative amount of synthesized fatty acid is reduced with pod-2 (gray) RNAi treatment compared to control (L4440) RNAi (black). This significant decrease demonstrates the effectiveness of the short-term adult-only RNAi and confirms our ability to measure synthesis with a 6-hour labeling period. Numbers shown represent the mean ± SEM, n = 5. Statistical significance was defined by t-tests (**p<0.01, ***p<0.001). (PDF) [file pone.0141850.s006.pdf]

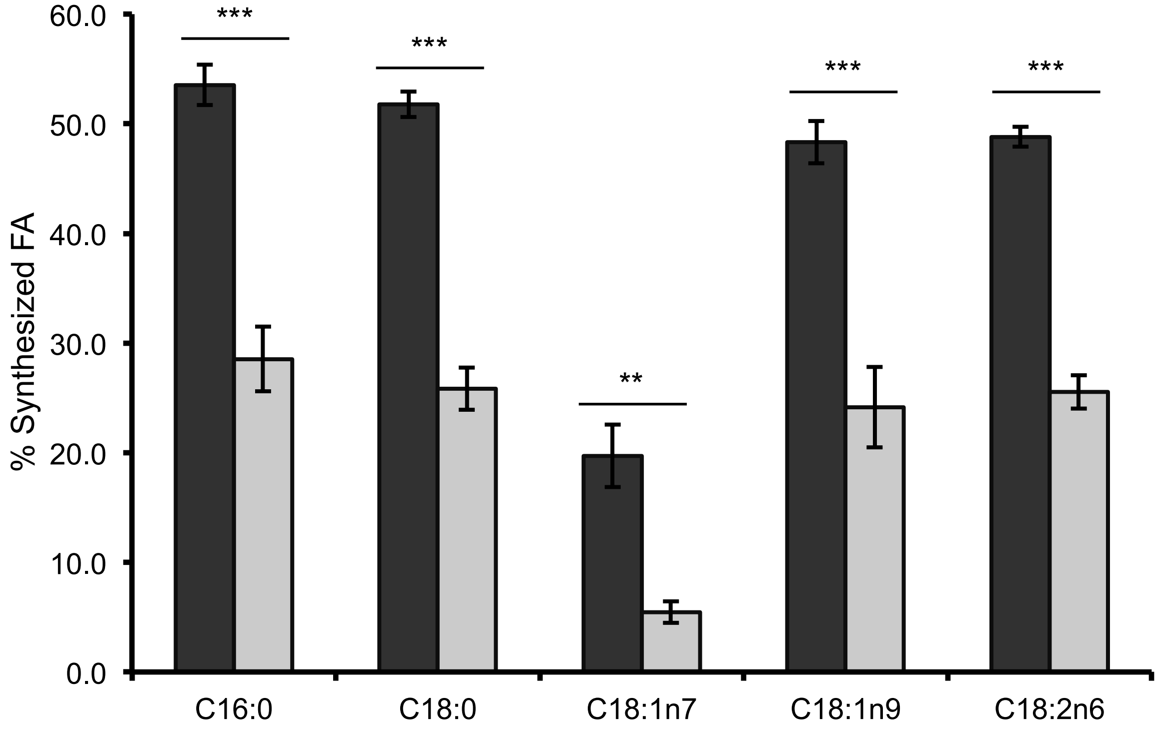

S4 Fig. Reduced Fatty Acid Synthesis in *pod-2* RNAi Treated Animals. The relative amount of synthesized fatty acid is reduced with *pod-2* (gray) RNAi treatment compared to control RNAi (black). This significant decrease demonstrates the effectiveness of the short-term adult-only RNAi and confirms our ability to measure synthesis with a 6-hour labeling period. Numbers shown represent the mean ± SEM, n=5. Statistical significance was defined by t-tests (**p<0.01, ***p<0.001).
