# Supplementary material for: 13C- and 15N-Labeling Strategies Combined with Mass Spectrometry Comprehensively Quantify Phospholipid Dynamics in C. elegans
Source: PLoS One. 2015 Nov 3;10(11):e0141850. doi: 10.1371/journal.pone.0141850 (PMC4631354; doi:10.1371/journal.pone.0141850)
Supplement: S5 Fig — To corroborate the decreased fatty acid incorporation seen after fat-7 RNAi treatment, we measured the amount of new fatty acids found in the phospholipid population of SCD mutant animals. Mutations in the fat-6 or the fat-7 desaturase have been shown to compensate for each other, and, because the RNAi against fat-7 also targets fat-6, we analyzed fat-6(tm331);fat-7(wa36) animals. These animals have very limited progeny production, and, therefore, we used fertile animals with very minimal larval contamination. There is significant developmental delay in fat-6;fat-7 nematodes, and there is not complete synchrony in the populations when assessed at day 3 of adulthood. Despite the technical challenges, the fat-6;fat-7 animals (purple) show significantly reduced fatty acid turnover in phospholipids when compared to fem-15;fer-1 control animals (black), similar to our observations with fat-7 RNAi. Numbers shown represent the mean ± SEM, n = 6. Unpaired t-tests established significance (*p<0.05, **p<0.01, ***p<0.001). (PDF) [file pone.0141850.s007.pdf]

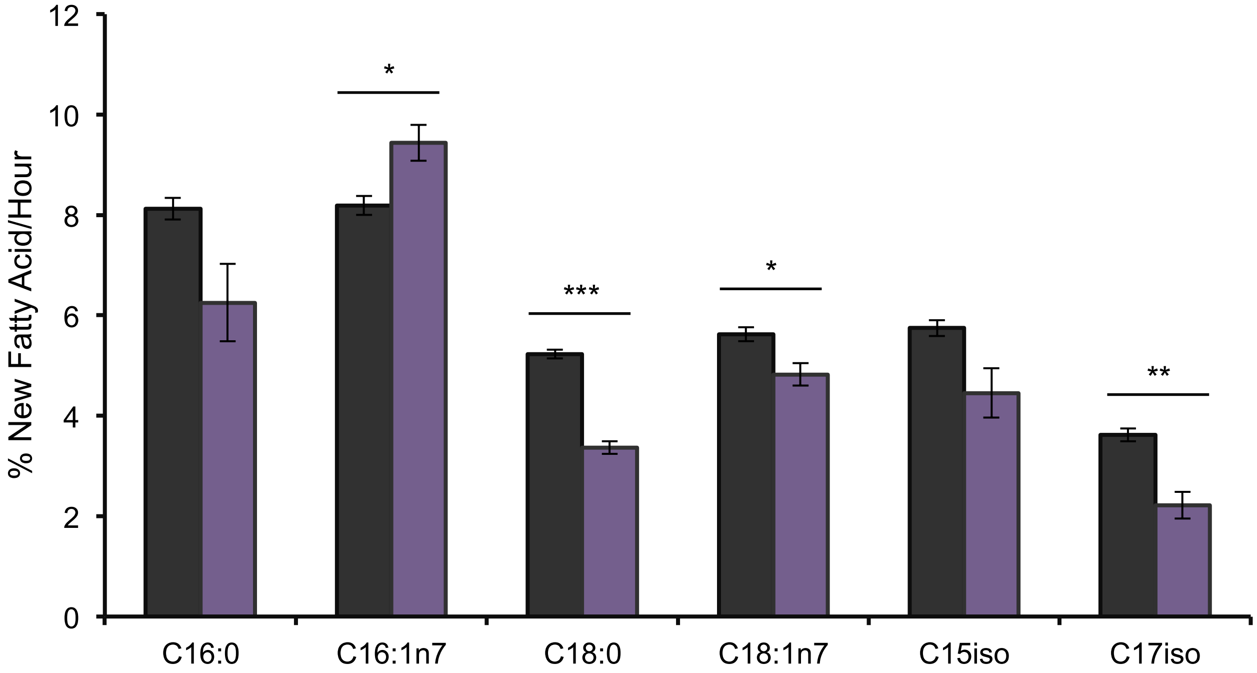

S5 Fig. ***fat-6;fat-7*** **Double Mutants Verify the Role of SCDs in Fatty Acid Replacement.** To corroborate the decreased fatty acid incorporation seen after *fat-7* RNAi treatment, we measured the amount of new fatty acids found in the phospholipid population of SCD mutant animals. Mutations in the *fat-6* or the *fat-7* desaturase have been shown to compensate for each other, and, because the RNAi against *fat-7* also targets *fat-6,* we analyzed *fat-6(tm331);fat-7(wa36)* animals. These animals have very limited progeny production, and, therefore, we used fertile animals with very minimal larval contamination. There is significant developmental delay in *fat-6;fat-7* nematodes*,* and there is not complete synchrony in the populations when assessed at day 3 of adulthood. Despite the technical challenges, the *fat-6;fat-7* animals (purple) show significantly reduced fatty acid turnover in phospholipids when compared to *fem-15;fer-1* control animals (black), similar to our observations with *fat-7* RNAi. Numbers shown represent the mean ± SEM, n=6. Unpaired t-tests established significance ($^{*}p<0.05$, $^{**}p<0.01$, $^{***}p<0.001$).
