# Supplementary material for: 13C- and 15N-Labeling Strategies Combined with Mass Spectrometry Comprehensively Quantify Phospholipid Dynamics in C. elegans
Source: PLoS One. 2015 Nov 3;10(11):e0141850. doi: 10.1371/journal.pone.0141850 (PMC4631354; doi:10.1371/journal.pone.0141850)
Supplement: S6 Fig — (A) Pharyngeal pump rates were measured for fat-5, fat-6, and fat-7 RNAi-treated animals to examine food intake. There was no significant change in food consumption in fat-5 or fat-7 RNAi-treated animals. There was a significant decrease in the pump rates of animals fed RNAi against fat-6 which may contribute the greater impact on overall membrane maintenance (see Fig 3C). Minimums of 35 animals were counted over three distinct experiments. (B) There is no significant change in fat storage as measured by the ratio of neutral lipid (NL) to phospholipid (PL) with any RNAi treatment. fat-6 RNAi-treated animals nearly met significance (p = 0.0506) which again highlights the more pronounced phenotype with this construct. Numbers shown represent the mean ± SEM, n = 3. Statistical significance was defined by t-tests (**p<0.01). (PDF) [file pone.0141850.s008.pdf]

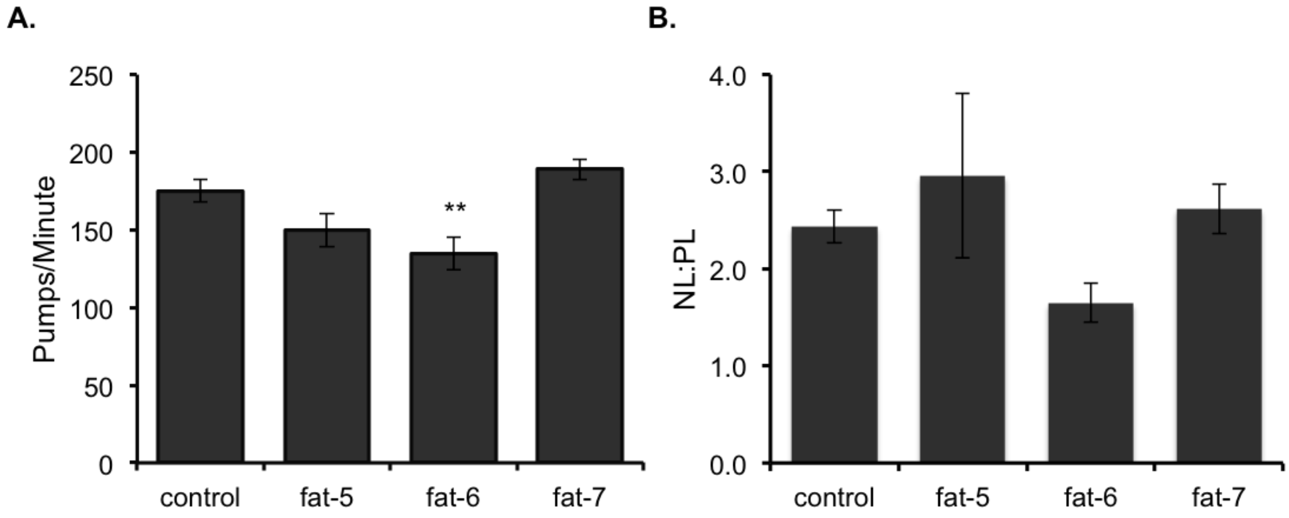

S6 Fig. Food Intake and Fat Storage are not Compromised with *fat-7* RNAi Treatment. (A) Pharyngeal pump rates were measured for *fat-5, fat-6,* and *fat-7* RNAi-treated animals to examine food intake. There was no significant change in food consumption in *fat-5* or *fat-7* RNAi-treated animals. There was a significant decrease in the pump rates of animals fed RNAi against *fat-6* which may contribute the greater impact on overall membrane maintenance (see Fig 3C). Minimums of 35 animals were counted over three distinct experiments. (B) There is no significant change in fat storage as measured by the ratio of neutral lipid (NL) to phospholipid (PL) with any RNAi treatment. *fat-6* RNAi-treated animals nearly met significance (p=0.0506) which again highlights the more pronounced phenotype with this construct. Numbers shown represent the mean ± SEM, n=3. Statistical significance was defined by t-tests (**p<0.01).
