# Supplementary material for: 13C- and 15N-Labeling Strategies Combined with Mass Spectrometry Comprehensively Quantify Phospholipid Dynamics in C. elegans
Source: PLoS One. 2015 Nov 3;10(11):e0141850. doi: 10.1371/journal.pone.0141850 (PMC4631354; doi:10.1371/journal.pone.0141850)
Supplement: S7 Fig — (A) Mass spectra can be used to identify the fatty acid tails associated with a given phospholipid population. Using the S2 scan from LC-MS/MS analysis, phosphatidylcholine (PtdCho) 38:7 shows the mass of two fragments (279.33, 301.25), indicating that the species consists of a C18:2 and a C20:5 fatty acid tail. (B) The MS2 scan of phosphatidylethanolamine (PtdEtn) 36:2 reveals two different species. Fragment masses 279.27, 281.33, and 283.40 correspond to fatty acid tails C18:2, C18:1, and C18:0. The MS2 scan shows that the PtdEtn 36:2 consists of one molecule with two C18:1 tails and another with C18:0 and C18:2 tails. (C) Table specifying fatty acid tail breakdown estimates for each listed PL species for both L4440 RNAi and fat-7 RNAi. (PDF) [file pone.0141850.s009.pdf]

A.

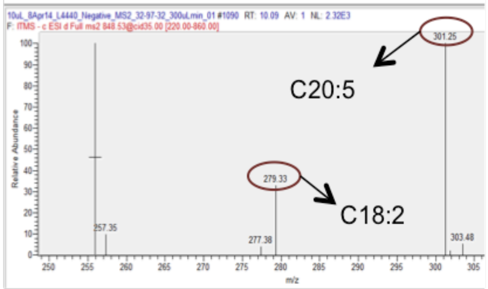

B.

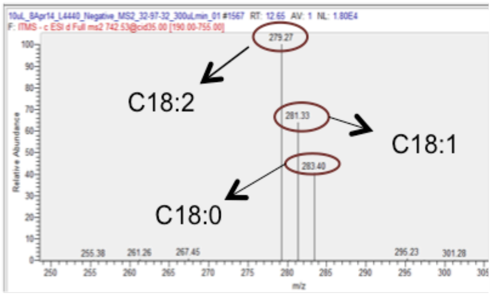

C.

Species	Control RNAi	*fat-7* RNAi
PtdCho 35:2	(18:2/17:0)60%<br>(18:1/17:1)25%<br>(20:2/15:0)15%	(18:1/17:1)90%<br>(19:1/16:1)10%
PtdCho 36:2	(18:1/18:1)70%<br>(19:1/17:1)30%	(18:1/18:1)70%<br>(18:0/18:2)30%
PtdCho 36:3	(18:2/18:1)70%<br>(20:3/16:0)25%<br>(18:3/18:0)5%	(18:3/18:0)60%<br>(18:2/18:1)30%<br>(20:3/16:0)10%
PtdCho 38:5	(20:4/18:1)70%<br>(20:5/18:0)30%	(20:5/18:0)85%<br>(20:4/18:1)15%
PtdCho 38:6	(20:5/18:1)100%	(20:5/18:1)100%
PtdCho 38:7	(20:5/18:2) 100%	(20:5/18:2)90%<br>(20:4/18:3)10%
PtdCho 40:8	(20:5/20:3)60%<br>(20:4/20:4)40%	(20:5/20:3)65%<br>(20:4/20:4)35%
PtdCho 40:9	(20:5/20:4)100%	(20:5/20:4)100%
PtdEtn 35:2	(18:2/17:0)60%<br>(18:1/17:1)25%<br>(20:2/15:0)15%	(18:1/17:1)90%<br>(19:1/16:1)10%
PtdEtn 36:2	(18:1/18:1)30%<br>(18:2/18:0)70%	(18:1/18:1)40%<br>(18:0/18:2)60%
PtdEtn 37:5	(20:5/17:0)100%	(20:5/17:0)100%
PtdEtn 38:5	(20:5/18:0)100%	(20:5/18:0)100%

S7 Fig. Assessment of Fatty Acid Tail Composition by LC-MS/MS. (A) Mass spectra can be used to identify the fatty acid tails associated with a given phospholipid population using the S2 scan from the LC-MS/MS analysis. For example, phosphatidylcholine (PtdCho) 38:7 shows the mass of two fragments (279.33, 301.25) indicating that the species consists of a C18:2 and a C20:5 fatty acid tail. (B) The MS2 scan of phosphatidylethanolamine (PtdEtn) 36:2 population reveals two different species. Fragment masses 279.27, 281.33, and 283.40 correspond to fatty acid tails C18:2, C18:1, and C18:0. The MS2 scan shows that the PtdEtn 36:2 consists of one molecule with two C18:1 tails and another with C18:0 and C18:2 tails. (C) Table specifying fatty acid tail breakdown estimates for each listed PL species for both control RNAi and *fat-7* RNAi.
