# Supplementary material for: 13C- and 15N-Labeling Strategies Combined with Mass Spectrometry Comprehensively Quantify Phospholipid Dynamics in C. elegans
Source: PLoS One. 2015 Nov 3;10(11):e0141850. doi: 10.1371/journal.pone.0141850 (PMC4631354; doi:10.1371/journal.pone.0141850)
Supplement: S8 Fig — (A) Scanning ion mode (SIM) profiles in control animals produced sufficient isotopomer abundance for both (i) C18:2n6 and (ii) C18:1n9 species. Therefore, we averaged the scans across the whole peak. (B) In contrast, the C18:2n6 SIM profile of fat-7 RNAi treated animals had inseparable C18:3 contamination in the certain scans. In order to minimize the isotopomer bias in this species, we manually examined each scan and excluded a scan from the C18:2n6 when the height of m/z 292 is over 75% of the m/z 294. For C18:1n9, scans were similarly excluded when scan height of m/z 294 is under 75% of m/z 296. (PDF) [file pone.0141850.s010.pdf]

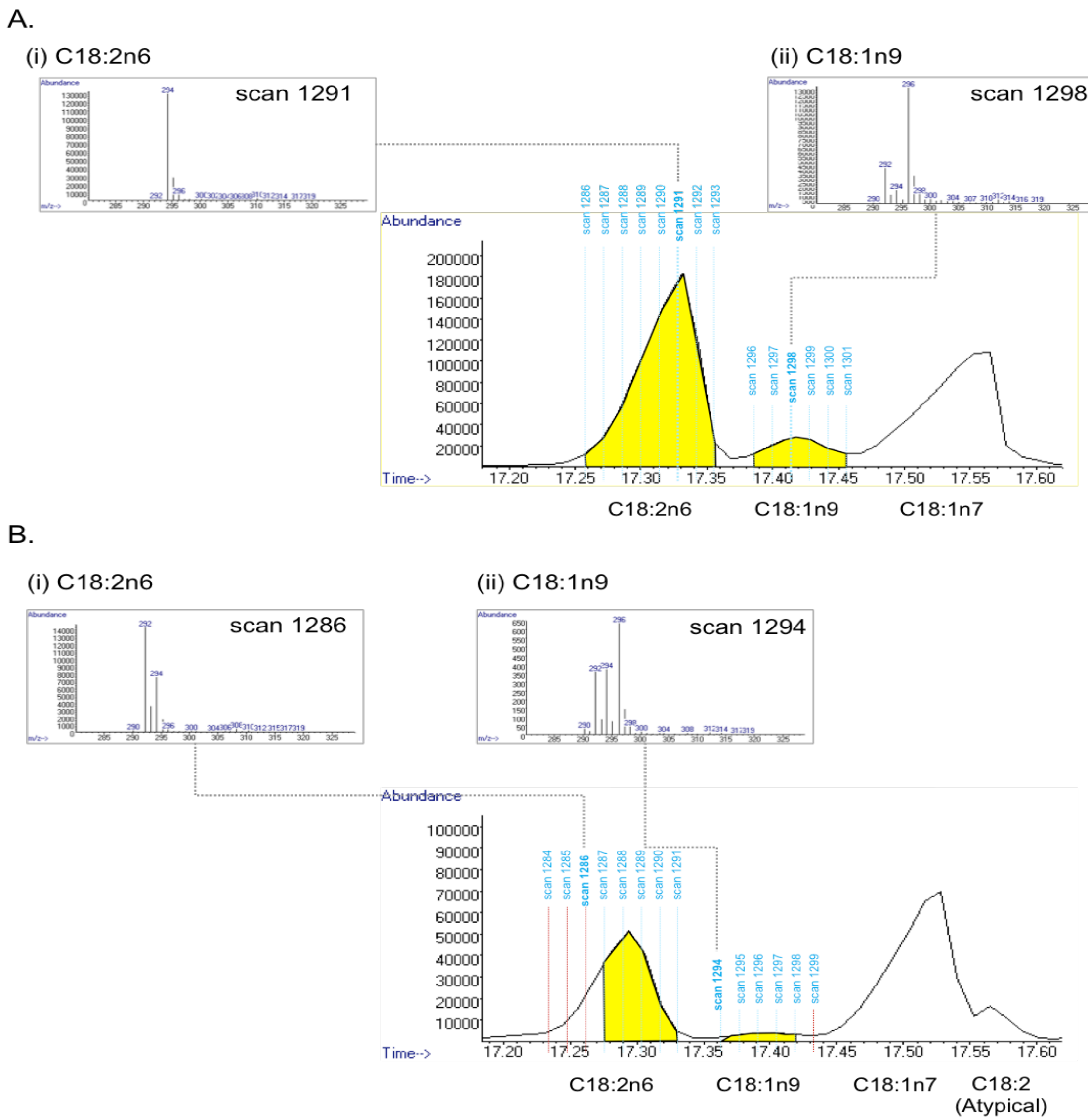

S8 Fig. Analysis of GC-MS Data in *fat -7* RNAi Treated Animals. (A) Scanning ion mode (SIM) profiles in control animals produced sufficient isotopomer abundance for both (i) C18:2n6 and (ii) C18:1n9 species. Therefore, we averaged the scans across the whole peak. (B) In contrast, the C18:2n6 SIM profile of *fat-7* RNAi treated animals had inseparable C18:3 contamination in the certain scans. In order to minimize the isotopomer bias in this species, we manually examined each scan and excluded a scan from the C18:2n6 when the height of m/z 292 is over 75% of the m/z 294. For C18:1n9, scans were similarly excluded when scan height of m/z 294 is under 75% of m/z 296.
