# Supplementary material for: 13C- and 15N-Labeling Strategies Combined with Mass Spectrometry Comprehensively Quantify Phospholipid Dynamics in C. elegans
Source: PLoS One. 2015 Nov 3;10(11):e0141850. doi: 10.1371/journal.pone.0141850 (PMC4631354; doi:10.1371/journal.pone.0141850)
Supplement: S1 Table — The relative abundance (%) of each fatty acid species of purified phospholipid (PL) and neutral lipid (NL) tails measured by gas chromatography. Data is presented as the average of 5 independent experiments ± SEM. (PDF) [file pone.0141850.s011.pdf]

	Control (l4440)		fat-7 RNAi	
**FA Species**	**PL**	**NL**	**PL**	**NL**
C14:0	<2%	3.6 ± 0.2	<2%	3.7 ± 0.2
C16:0	5.7 ± 0.3	6.7 ± 0.5	4.1 ± 0.2	6.5 ± 0.5
C18:0	8.0 ± 0.5	4.1 ± 0.2	16.0 ± 0.6	13.8 ± 1.1
Total SFA	14.9	14.4	20.9	23.9
C16:1n7	2.0 ± 0.1	5.8 ± 0.2	2.4 ± 0.1	5.9 ± 0.3
C18:1n9	4.5 ± 0.1	11.3 ± 0.2	<2%	3.8 ± 0.6
C18:1n7	20.3 ± 0.6	18.4 ± 0.4	21.9 ± 0.7	17.1 ± 1.0
Total MUFA	26.9	35.5	25.4	26.8
C18:2n6	10.0. ± 0.3	7.5 ± 0.2	4.3 ± 0.5	4.1 ± 0.3
C20:3n6	4.9 ± 0.2	<2%	2.9 ± 0.1	<2%
C20:4n6	3.8 ± 0.2	<2%	2.5 ± 0.2	<2%
C20:4n3	5.3 ± 0.2	<2%	4.9 ± 0.4	<2%
C20:5n3	19.4 ± 0.3	4.4 ± 0.2	24.9 ± 0.7	4.0 ± 0.2
Total MUFA	43.5	15.1	39.4	9.7
C15iso	2.3 ± 0.1	6.5 ± 0.5	<2%	7.5 ± 0.6
C17iso	4.3 ± 0.5	6.4 ± 0.7	3.3 ± 0.4	6.6 ± 0.7
Total mmBCFA	6.7	12.9	5.1	14.1
C17D	3.5. ± 0.2	15.9 ± 0.4	4.7 ± 0.3	18.7 ± 0.7
C19D	<2%	5.1 ± 0.2	2.4 ± 0.2	5.5 ± 0.4
Total CFA	5.3	21.0	7.2	24.2

S1 Table. Fatty Acid Tail Composition of day 3 *fem-1;fer-15* on RNAi. The relative abundance (%) of each fatty acid species of purified phospholipid (PL) and neutral lipid (NL) tails measured by gas chromatography. Data is presented as the average of 5 independent experiments ± SEM.
